# Supplementary material for: Effect of radiofrequency and pelvic floor muscle training in the treatment of women with vaginal laxity: A study protocol
Source: PLoS One. 2021 Nov 9;16(11):e0259650. doi: 10.1371/journal.pone.0259650 (PMC8577744; doi:10.1371/journal.pone.0259650)
Supplement: S1 File — (DOCX) [file pone.0259650.s002.docx]

**University of Campinas**

**School of Medical Sciences**

**Effect of Fractionated Microablative Radiofrequency and Pelvic Floor Muscle Training in the Treatment of Women with Vaginal Laxity Complaint: Randomized Clinical Trial**

**RESEARCH PROJECT - DOCTORATE**

**PRINCIPAL RESEARCHERS: Prof. Dr. Luiz Gustavo Oliveira Brito**

**Profa. Dra. Cássia Raquel Teatin Juliato**

**COLLABORATING RESEARCHER: Gláucia Miranda Varella Pereira**

**I authorize this research:**

_______________________________________

*Profa. Dra. Lucia Helena S. Costa Paiva*

Gynecology Division of Centro de Atenção à Saúde Integral da Mulher – CAISM/UNICAMP

**Campinas**

**10/04/2019**

**PROJECT IDENTIFICATION**

Title: Effect of Fractionated Microablative Radiofrequency and Pelvic Floor Muscle Training in the Treatment of Women with Vaginal Laxity Complaint: Randomized Clinical Trial

Executing institution / proponent: State University of Campinas - UNICAMP - Faculty of Medical Sciences - Department of Tocogynecology - Division of Gynecology

Place of development of the proposal: Urogynecology outpatient clinic at the School of Medical Sciences and the Physiotherapy outpatient clinic at Center for Integral Attention to Women's Health - CAISM of the State University of Campinas - UNICAMP.

RESEARCHER IDENTIFICATION

Principal researcher: Prof. Dr. Luiz Gustavo Oliveira Brito

Position / Function: Professor at the Department of Tocogynecology at the Faculty of Medical Sciences - UNICAMP / SP.

RG: 79.972.797 - 0

CPF: 833. 347. 693 - 72

Address: State University of Campinas, Faculty of Medical Sciences, Department of Tocogynecology - Center for Integral Attention to Women's Health (CAISM), Rua Tessália Vieira de Camargo, 126. Cidade Universitária “Zeferino Vaz”. CEP-13083-887 - Campinas, SP - Brazil.

E-mail: lgobrito@gmail.com / lgobrito@unicamp.br

Contact phone: 19-3521-9595

Lattes platform curriculum: http://lattes.cnpq.br/0402956055809744

**Signature:**____________________________________________________________

Principal Researcher: Prof. Dra. Cássia Raquel Teatin Juliato

Position / Function: Gynecologist, Professor at the Department of Tocogynecology at the Faculty of Medical Sciences - UNICAMP / SP.

RG: 25. 311. 099 - 3

CPF: 255. 104. 518 - 58

Address: State University of Campinas, Faculty of Medical Sciences, Department of Tocogynecology - Center for Integral Attention to Women's Health (CAISM), Rua Tessália Vieira de Camargo, 126. Cidade Universitária “Zeferino Vaz”. CEP-13083-887 - Campinas, SP - Brazil.

E-mail: cassia.raquel@gmail.com

Contact phone: (19) 35219516

Lattes platform curriculum: <http://lattes.cnpq.br/2246374748063899>

**Signature:**____________________________________________________________

Collaborating researcher: Gláucia Miranda Varella Pereira

Position / Function: Master Physiotherapist in Women's Health and PhD student at the Post-Graduation Course in Tocogynecology at the State University of Campinas - UNICAMP / SP.

RG: 12.232.338

CPF: 046.330.266 -40

Address: State University of Campinas, Faculty of Medical Sciences, Department of Tocogynecology - Center for Integral Attention to Women's Health (CAISM), Rua Tessália Vieira de Camargo, 126. Cidade Universitária “Zeferino Vaz”. CEP-13083-887 - Campinas, SP - Brazil.

E-mail: glauciavarella@gmail.com

Contact phone: (37) 99997 7771

Lattes platform curriculum: <http://lattes.cnpq.br/8072128386816527>

**Signature:**____________________________________________________________

**Abstract**

**Introduction:** Vaginal laxity, a condition rarely discussed among patients and doctors, is defined as a complaint of excess vaginal flaccidity. It has a prevalence of 24% and seems to be associated with young age, vaginal births, symptoms of prolapse, and objective prolapse, being, therefore, a somatic and non-psychogenic dysfunction. A recent study showed associations between the hiatal, genital hiatus, and perineal body areas, suggesting that vaginal laxity is a manifestation of the hyperdistensibility of the levator ani. Women with vaginal flaccidity may be representative of an early stage in the development of pelvic organ prolapse; however, this has not been assessed previously. A standardized definition and means for consulting patients regarding such symptoms do not yet exist. Surgical procedures for vaginal laxity with posterior repair/perineoplasty are more commonly recommended, however, there are risks of dyspareunia. Non-surgical and lower-cost options can contribute to the treatment of vaginal laxity. Among them, the training of the pelvic floor muscles and radiofrequency standout. To date, no clinical trial has been developed to assess the role of pelvic floor muscle and radiofrequency training in vaginal laxity.

**Objectives:** To compare the effect of isolated fractional microablative radiofrequency and isolated pelvic floor muscle training in women with complaints of vaginal laxity.

**Methodology:** This is a clinical, randomized, prospective, controlled, parallel, and non-blind study. The research will be carried out at the outpatient clinic for surgical gynecology at the State University of Campinas - UNICAMP / SP. The study will include pre-menopausal women aged ≥ 18 years, vaginal delivery, with complaints of vaginal laxity assessed by direct question (yes/no) and by the Vaginal Laxity Questionnaire and with availability to attend therapies on the scheduled date and places to carry out the proposed treatment. Exclusion criteria are the use of a pacemaker; decompensated heart disease; cognitive deficit; peripheral or central neurological disorders; the presence of any type of cancer; the presence of cervical dysplasia; the history of active urinary or vaginal infection; decompensated metabolic diseases; physical therapy treatment with previous pelvic floor training in the last 12 months; use of estrogen via vaginal or oral hormonal therapy in the last 6 months, patients who have already undergone prolapse or sling correction surgery; the presence of pelvic organ prolapse stage 2 onwards**.** The selected participants will be divided into 2 groups of treatment protocols: group 1 - radiofrequency and group 2 - training of the pelvic floor muscles. After the intervention period, the groups will be reevaluated in 30 and 180 days. For the sample calculation, we used values of sexual function assessed using the Female Sexual Function Index questionnaire. When considering a study power of 80%, an alpha of 0.05 with the two-tailed test, it was verified that the minimum number of participants required in each group will be added to a percentage of 30% of loss in the sample, totaling 68 women, 34 in each group (isolated radio frequency and isolated pelvic floor muscle training).

A descriptive analysis of the data will be performed to characterize the research participants, in the form of values ​​of absolute frequency and percentage (relative) for categorical variables and values ​​of mean and standard deviation for numerical variables. Then, statistical analysis of comparison and correlation of the data obtained from the following statistical tests will be performed: Kolmogorov-Smirnov to assess the normality of the sample. Depending on the results obtained in the normality test, the Analysis of Variance (if the data shows normal distribution) or the Wilcoxon and Mann-Whitney Test (if the data are non-parametric) will be used for comparative analyzes between the groups. Likewise, Pearson or Spearman tests will be used for correlational analyzes. Categorical variables will be analyzed using the chi-square test or Fisher's exact test. Statistical analyzes will be performed using the statistical program SPSS (Statistical Package for the Social Sciences), adopting a significance level of 5% (p <0.05).

The data will also be evaluated using the method of analysis of variance for repeated measures (ANOVA for repeated measures) with the objective of simultaneously verifying the influence of the 2 study groups (intergroup evaluation) and of the 2 evaluations (intragroup evaluation) for each one. variables, in order to obtain the estimation of the group x time interaction effect. If the numerical variables do not have a normal distribution, they will be transformed into ranks or posts.

**Keywords:** radiofrequency, vaginal laxity, physical therapy, pelvic floor muscles training, sexual dysfunction; randomized controlled trial

**SUMMARY**

1. INTRODUCTION7
2. JUSTIFICATION12
3. OBJECTIVES13

3.1 General Objective13

3.2 Specific Objectives13

1. HYPOTHESIS14
2. MATERIALS AND METHODS15

5.1 Study Design15

5.2 Sample Size15

- 1. Study Setting and Eligibility Criteria15
     1. Inclusion Criteria16
     2. Exclusion Criteria16
  2. Variables and Concepts17
     1. Independent Variables17
     2. Dependent Variables17
     3. Descriptive Variables19
  3. Treatment, techniques, tests and/or exams20
     1. Treatment Protocol20
     2. Assessment Questionnaires21
     3. Functional and Morphometric Assessment of the Pelvic Floor24
  4. Data Collection.....................................................................................................25

5.7 Participants Follow-up.........................................................................................26

5.8 Criteria for discontinuing, suspending, and/or terminating the research26

5.9 Quality control.....................................................................................................26

5.10 Data analysis27

5.11 Dissemination of results....................................................................................27

1. ETHICAL ASPECTS 28
2. BUDGET 29
3. ACTIVITY PLAN AND SCHEDULE 30
4. REFERENCES31

10. **RESEARCH DISSEMINATION...........................................................................36**

APPENDIX 37

**1-INTRODUCTION**

**Definition and risk factors**

Vaginal laxity is defined by the International Urogynecology Association (IUGA) and the International Continence Society (ICS) as a complaint of excess vaginal flaccidity^1^. There is no consensus regarding a standard definition for this clinical situation and there has been an increase in demand for treatment of vaginal laxity, especially in the area related to genital aesthetics ^2, 3^.

This condition is rarely discussed among patients and their doctors, possibly due to the lack of evidence-based treatments, embarrassment and lack of knowledge in recognizing this condition^4^. In the opinion of urogynecologists, vaginal laxity still presents itself as an underreported condition with reports of discomfort that can affect sexual function and relationships ^5, 6^.

The prevalence of vaginal laxity is 24% and seems to be associated with young age, vaginal births, symptoms of prolapse and objective prolapse, being, therefore, a somatic and non-psychogenic dysfunction^7^. Other risk factors are fetal macrosomia, history of instrumental delivery (forceps), multiparity and changes in connective tissues. The way women perceive their genitalia has a strong and positive impact on their sexual function ^8^.

It is speculated that pregnancy and childbirth play a role in vaginal laxity ^5^. Although there is no proven link between vaginal laxity and childbirth, research indicates that vaginal delivery can result in pelvic floor injury ^7, 9^. Trauma to the pelvic floor and vagina during pregnancy and vaginal delivery can lead to the lengthening of the vaginal opening leading to permanent changes in sexual and physical sensitivity during intercourse. These changes promote an important reduction in the quality of life of women and their partners^10, 11^.

Potential consequences associated with vaginal delivery that extend beyond the postpartum period are: urinary incontinence, pelvic organ prolapse, chronic pelvic pain and sexual dysfunction ^12-15^. Not all women adapt to the psychological and physical changes in the postpartum period, which can lead to changes in the affective relationship with the partner ^16^. Two-thirds of women experienced significant worsening of sexual function six months after vaginal delivery^17^. Klein *et al.* reported that women without perineal trauma were more likely to return to sexual activity at six weeks postpartum compared to women with perineal trauma ^18^. In addition, dyspareunia is reported by 41 to 67% of women between two and three months after delivery ^12, 19-21^.

Both vaginal delivery and trauma to the levator ani muscle are associated with an increase in the diameter of the genital hiatus^22^. Avulsion of the levator ani muscle, especially if proven bilaterally, would have some effect on female sexual function ^23^. During vaginal delivery, the puborectal muscle is exposed to a high degree of stretching, with an estimated stretch ratio of 1.5 to 3.5^24, 25^. The degree of muscle stretching seems to vary from 25 to 250%^25^. Muscle physiology studies have shown that substantial, macro and microscopic injury can occur if the skeletal muscle fiber is stretched to more than 1.5 times its original extension ^26^. It is not surprising, therefore, that 10-35% of women have a traumatic lesion of the puborectal muscle at their bone insertion^27-29^. This results in an increase in the hiatus of 20-30%,^30^ and a more distensible and less contractile pelvic floor muscle ^22^. In a study on peripartum change in hiatal dimensions, more than 28% of primiparous women were diagnosed with irreversible hiatal hyperdistension or "lifter microtrauma" at 4 months postpartum, regardless of avulsion,^31^ and with no evidence of cure after two years of follow-up ^32^. The genital hiatus is limited by the puborectal muscle, a component of the levator ani muscle, and plays an important role in defining the high vaginal pressure zone ^33^.

In a study that evaluated more than 300 women with vaginal laxity, associations were found between the hiatal area, the genital hiatus and the perineal body during the Valsalva maneuver, suggesting that vaginal laxity is a manifestation of the hyperdistensibility of the levator anus and not of the vagina^7^. The measurements of the levator ani gap are strongly associated with the genital gap and the perineal body measured by the POP-Q instrument and, therefore, it is not surprising that the latter parameter was also strongly associated with the symptom of vaginal laxity^7^. Women with vaginal flaccidity may be representative of an early stage in the development of pelvic organ prolapse; however, this has not been evaluated previously^5^. A standardized definition and means for consulting patients regarding such symptoms do not yet exist ^5^.

Serum estradiol levels in women of reproductive age range from 30 to 300 pg / mL, depending on the phase of the menstrual cycle. Postmenopausal women have this level reduced by more than 90% to an average of 6.5 pg / mL ^34^. Deep changes occur in the vulvovaginal and urogenital mucosa with the loss of estrogenic stimulation ^35^. Hypoestrogenism also results in changes in connective tissue, changes in pelvic structure and decline in collagen quality ^36^. Age and hormonal changes cause deterioration and relaxation of connective tissue and collagen fibers, decreasing the support of pelvic organs due to the decrease in diameter and the number of striated periurethral muscle fibers and the pelvic floor^37^. This pathophysiology is important for understanding some types of treatment, such as radiofrequency.

**Diagnosis and treatment**

The reduction in vaginal sensation during sexual intercourse may be related to anatomical damage to the perineal body, prolapse in stage 1, laxity of the vaginal canal or introitus, underlying damage to nerves and connective tissue during pregnancy and childbirth or, potentially, a combination of these factors ^38^.

The diagnosis of vaginal laxity is based on the patients' self-report. A comprehensive medical history, physical examination and psychosexual assessment are the initial steps to properly identify patients with vaginal laxity ^39^.

An instrument that has been used in clinical research to assist in the identification and degree of laxity is the “Vaginal Laxity Questionnaire”. This self-reported assessment of vaginal laxity uses a seven-point scale associated with a question: How would you rate your current level of vaginal laxity? or laxity during intercourse?^40^.

Anatomy plays an important role in understanding the different structures involved in pelvic support ^41^. The pelvic girdle is composed of several layers of supporting muscles and fascias that interconnect and overlap, contributing to the overall support and normal functioning of the vagina and its adjacent structures ^42, 43^. History and physical examination will determine whether the patient is a candidate for vaginal procedures or a more complex vaginal reconstruction approach ^44^. Before these patients can be properly managed, it is important to understand the complex structural mechanics of posterior vaginal wall failure ^45^. Failure of the posterior wall may involve failure of the support of the perineal body and the levator ani muscles, which can result in an enlarged genital hiatus ^45^. The levator muscles provide a tonic and cephalic action that keeps the genital gap closed to a normal dimension in response to pressure. If the levators are weakened or injured, or if the fascial attachments of the posterior vaginal wall are affected (rectocele), a descending descent of the perineal body occurs and the hiatus opens. ^45^. The weakening of the endopelvic fascia in the anterior compartment could be further studied to associate urethral hypermobility and consequent stress urinary incontinence with vaginal laxity^46^.

The diagnosis of pelvic organ prolapse requires clear clinical evidence, starting with a woman with symptoms related to "downward displacement" of a pelvic organ. Symptoms are usually worse in situations where gravity can worsen prolapse (for example, after long periods of standing or exercising) and better when gravity is not a factor, for example, lying in a supine position. Again, the symptoms may be more noticeable at times of abdominal effort, for example, defecation ^47^.

The function and contractility of the pelvic floor muscles are assessed using the Oxford Scale Modified by Laycock (1994)^48^. This scale is classified from zero to 5, being zero-without objective perineal function and 5- optimal contraction of intensity and cranial elevation of the vaginal wall ^48^.

Surgical procedures for vaginal laxity with posterior repair / perineoplasty are more commonly recommended, however, 83% of the interviewed urogynecologists reported a potentially important concern with cases of dyspareunia ^5^. In recent years there has been an increasing number of various types of vulvovaginal surgeries marketed as ways to improve appearance or sexual gratification. Among them, the so-called vaginal rejuvenation, designer vaginoplasty, revirginization and G-spot amplification stand out. Some procedures, such as vaginal rejuvenation, seem to be modifications of traditional vaginal surgical procedures. Other procedures are performed to change the size or shape of the major or minor lip. Revirginization involves hymenal repair in an attempt to approximate the virginal state. Amplification of the G-spot involves the injection of collagen into the anterior wall of the vagina. Despite being performed, the safety and effectiveness of these procedures in the long term have not yet been documented ^49^.

A non-surgical option for treating vaginal laxity includes training the pelvic floor muscles that was initially recommended for the treatment of urinary incontinence ^4^. The function of the pelvic floor muscle appears to play an important role in female sexual function, and contraction of the levator ani muscle appears to increase sexual response^50^.

The contraction of the pelvic floor muscles also plays an important role in the female orgasmic response. Women with weak muscles who receive pelvic floor rehabilitation and strengthen the muscles in that region perceive a positive effect on their sex life ^51^. Pelvic floor muscle training (PFMT) has proven to be an effective treatment for dyspareunia ^52^. It is generally recommended as a first-line treatment, since it has been associated with minimal adverse effects and low cost^53^.

Another non-surgical therapeutic possibility to treat vaginal laxity is radiofrequency (RF). A pilot study for the use of radiofrequency for the treatment of vaginal laxity showed that the treatment was well tolerated by the participants and showed subjective improvement in vaginal narrowing, sexual function and decreased sexual discomfort^4^.

  Radiofrequency is generated by the electric field resulting from the oscillation of the electric current, which, in turn, induces the translational movement of charged atoms and molecules and hinders the rotation of polar molecules^54^. This moving molecule is largely responsible for the heat capacity and the increase in local temperature. In the presence of an electric field, the molecules orient themselves along the direction of the field, but due to the viscosity of the water, energy is needed to rotate the dipoles resulting in energy transfer to the tissue. The resistance or impedance converts electrical current to thermal energy generating heat in relation to the amount of current time and exposure. Consequently, energy is dispersed in three-dimensional volumes of tissue at controlled depths ^54^.

Radio frequency energy has a long history of use in the mucous tissue of the vagina and skin ^55-57^. Through the creation of heat via impedance as the electrical current is conducted through the vaginal tissue, fibroblast stimulation occurs and the therapeutic result is achieved ^58^. The temperature range of the target tissue is between 40º and 47 ℃. The effectiveness of radiofrequency in natural moisture was demonstrated in the histological study of radiofrequency in the vaginal tissue of sheep ^59^. Radio frequency was also effective for vulvovaginal rejuvenation ^4^. A study using low-energy radio frequency for introital vaginal laxity in pre-menopausal women pointed out improvements in both laxity and sexual function. The effects were maintained for 12 months and no adverse events were reported ^10^.

To date, no clinical trial has been developed to assess the role of pelvic floor muscle and radiofrequency training in vaginal laxity.

**2. JUSTIFICATION**

Surgical procedures for the repair of vaginal laxity are commonly recommended but can lead to dyspareunia, an important adverse factor in female sexual function ^5^. Other surgical procedures are indicated, such as revirginization surgery with hymenal repair, however, the safety and efficacy of this treatment has not yet been documented.

Non-surgical options that offer minimal adverse effects can be indicated for the treatment of vaginal laxity at lower costs than surgical procedures. The training of the pelvic floor muscles is presented as the first line in the treatment of urinary incontinence. Radiofrequency, on the other hand, showed improvements in both vaginal laxity and sexual function maintained for 12 months in a few studies previously discussed. Compared to other conservative treatments such as laser, RF has more economic costs, and the possibility of reaching populations with less purchasing power to be able to treat.

Thus, when evaluating the behavior of both the radio frequency and the training of the pelvic floor muscles in vaginal laxity, we can contribute to the indication of these modalities to improve the quality of female sexual life.

**3. OBJECTIVES**

- 1. **General Objective**

To compare the effect of isolated fractional microablative RF and isolated PFMT in women with vaginal laxity symptoms.

**3.2 Objetivos Específicos**

• Compare the effect of isolated RF and isolated PFMT on sexual function.

• Compare the effect of isolated RF and isolated PFMT on urinary symptoms.

• Compare the effect of isolated RF and isolated PFMT on the contractility and function of pelvic floor muscles.

• Compare the effect of isolated RF and isolated PFMT on the vaginal laxity scale.

• Compare the effect of isolated RF and isolated PFMT on levels of vaginal symptoms and distress in women with vaginal laxity.

**4. HYPOTHESES**

• Women undergoing RF treatment should experience subjective improvement non-inferior than women treated with PFMT for vaginal laxity.

• The quality of life of women with both treatments will be similar.

• RF treatment will have adverse effects compared to PFMT; the latter is usually free of side effects.

• RF treatment will improve sexual function in women with vaginal laxity in a similar way with PFMT.

**5. MATERIALS AND METHODS**

**5.1 Study Design**

Clinical, randomized, prospective and controlled, parallel, non-inferiority, non-blind study.

**5.2 Sample Size**

The sample calculation was based on the study of Krychman *et al*. ^40^, which demonstrated that RF therapy was associated with significant clinical and statistically significant improvement in sexual function in women with vaginal laxity, when data analysis was performed in a group containing 73 patients. To calculate the sample of the present study, we used values of sexual function assessed using the FSFI questionnaire. There was an increase of 7 points in the FSFI score in the group treated with radio frequency and an increase of 3 points in the control group. When considering a study power of 80%, an alpha of 0.05 with two-tailed test, it was found that the minimum number of participants required in each group will be added to a percentage of 30% loss in the sample, totaling 68 women, 34 in each group (isolated RF and isolated PFMT).

Randomization will be carried out through a computer program, in a 1: 1 ratio, in two blocks. The numbers corresponding to the study groups (1. Radiofrequency Group and 2. Pelvic Floor Muscle Training Group) will be placed in opaque envelopes that will be opened by the woman after signing the consent form and undergoing pre-intervention evaluation.

**5.3 Study Setting and Eligibility Criteria**

The research will be developed at the urogynecology outpatient clinic at the School of Medical Sciences and the Physiotherapy outpatient clinic at Center for Integral Attention to Women's Health - CAISM of the State University of Campinas – UNICAMP.

The selection of volunteers will be carried out by referring women with clinical diagnosis of vaginal laxity from the urogynecology outpatient Clinic and the Center for Attention to Integral Health of Women - CAISM / UNICAMP and also through the dissemination of the research through social media and printed ads for spontaneous demand from volunteers from February 2020 to June 2021. Through lectures, all women will be informed about the analysis procedures, criteria and treatment to which they will be submitted, including the possibility of not participating in the study. Those who accept to participate must sign a Free and Informed Consent Form, approved by the Research Ethics Committee (Appendix 10).

5.3.1 Inclusion Criteria:

Women aged ≥ 18 years, vaginal delivery, complaining of vaginal laxity assessed by direct question (yes / no) and by the Vaginal Laxity Questionnaire (VLQ) (Appendix 1), and willing to attend therapies on the date and locations scheduled to carry out the proposed treatment.

5.3.2. Exclusion Criteria:

Both groups will exclude those with:

- Use of pacemaker;

- Decompensated heart disease;

- Cognitive deficit;

- Peripheral or central neurological disorders;

- Presence of any type of cancer;

- Presence of cervical dysplasia;

- History of active urinary or vaginal infection

- Decompensated metabolic diseases;

- Patients undergoing physical therapy with pelvic floor training;

- Patients using estrogen via vaginal / oral in the last 6 months;

- Patients who have already undergone prolapse or sling correction surgery.

- Patients with pelvic organ prolapse stage 2 onwards;

- Degree of force of contraction of the pelvic floor muscles equal to zero according to the Modified Oxford Scale.

**5.4 Variables and concepts**

5.4.1 Independent variables (intervention groups):

- Application of Radiofrequency: application of microablative radiofrequency via vaginal with conversion to thermal energy that aims at the degradation of collagen fibers and new modeling of these promoting strengthening of the support structures of the pelvic floor.

- Pelvic Floor Muscle Training (PFMT): physiotherapy treatment aimed at strengthening the support structures of the pelvic floor.

- Global Response Assessment: this subjective scale will be adapted for the present study according to the study by Millheiser *et al*. ^4^ This is a seven-point scale with an answer to the following question: How are you feeling now (levels of vaginal laxity / tightness and sexual satisfaction) compared to starting treatment? 1-markedly improved, 2-moderately improved, 3-slightly improved, 4-no change, 5-slightly worse, 6-moderately worse, 7-much worse.

5.4.2 Dependent variables:

- *Vaginal laxity:* clinical condition related to the feeling of “laxity” of the vaginal opening perceived by the woman and / or sexual partnership during penetration5. Vaginal laxity will be assessed clinically, by means of a direct question (yes / no) and by the Vaginal Laxity Questionnaire (VLQ) (Appendix 1).

*- Female sexual function:* clinical conditions of women linked to the sexual act investigated through the validated Female Sexual Function Index (FSFI) questionnaire ^60^ (Appendix 2). Dyspareunia will be assessed using the Marinoff Scale ^61^ (Appendix 3).

*- Vaginal symptoms:* clinical conditions related to the investigated vagina using the questionnaire validated by the International Consultation on Incontinence Questionnaire - Vaginal Symptoms (ICIQ-VS)^62^ (Appendix 4).

- *Sexual distress and depression*: sexual suffering will be measured by the Female Sexual Distress Scale-Revised scale ^63^ (Appendix 5).

*- Urinary incontinence (UI):* urinary loss during stress situations (such as coughing, sneezing, jumping); investigated using the International Consultation on Incontinence Questionnaire Short Form (ICIQ UI-SF), which allows qualifying and quantifying urinary loss in addition to investigating its impact on quality of life^64^ (Appendix 6).

- *Urethral mobility:* difference in the positioning of the urethra at rest and during the Valsalva maneuver during clinical evaluation.

*- Pelvic organ prolapse (POP):* descent of the anterior and / or posterior vaginal wall, as well as the vagina apex (uterus or vaginal dome after hysterectomy), investigated using the Pelvic Organ Prolapse Quantification (POP-Q) instrument for evaluation and prolapse staging^47^ (Appendix 7 ).

- *Pelvic floor muscle strength and function:* intensity of pressure that the perineal musculature exerts on the examiner's two fingers during vaginal touch. Patient positioned in supine position with feet supported will be instructed to perform a maximum voluntary muscle contraction, graded by the Modified Oxford Scale, in 5 levels: zero or absent = no signs of muscle contraction are observed; 1 = minimal muscle strength (signs of discreet contractility, without joint movements); 2 = weak muscle strength (mobility in all normal directions, eliminating gravity); 3 = regular muscle strength (movements of normal amplitude against the action of gravity); 4 = good muscular strength (full mobility against the action of gravity and a certain degree of resistance); 5 = normal muscle strength (complete mobility against severe resistance and against the action of gravity)^48^ (Appendix 8). The morphometry of the pelvic floor muscles will be performed using the transperineal ultrasound device. The measurements will be performed at rest, Valsalva maneuver and muscle contraction of the pelvic floor with the patient in the lithotomy position. The indications for this evaluation method are in accordance with Dietz *et.al*^65^ (Appendix 8).

- *Vaginal Thickness:* The vaginal thickness will be assessed in its proximal, middle, and distal third using two approaches - transabdominal and transvaginal^66, 67^. The probes 4C-D 2 at 5 MHz transabdominal and 5 to 9 MHz transvaginal will be used (Appendix 8).

5.4.2 Descriptive variables:

- Age: expressed in absolute number, in years, informed by the patient;

- Skin color: skin color declared by the woman herself and categorized as white and not white;

-Education level: degree of study of the patient specified in complete or incomplete elementary school, complete or incomplete high school and complete or incomplete higher education;

- Frequency of sexual intercourse with penetration: weekly frequency of sexual intercourse with vaginal penetration presented in absolute numbers;

- Smoking: patient with addiction to any type of tobacco, self-declared smoker, ex-smoker or non-smoker;

- Parity: number of pregnancies reported by the woman and expressed in absolute numbers, differentiating vaginal delivery, cesarean delivery and abortion.

- Menopause: age, in years, of the last menstrual period.

- Body mass index (BMI): measure of the body mass index, by calculating the ratio between the weight and the square of the patient's height, classified below (<20); normal (20-25); overweight (26-30); moderate obesity (31-35); severe obesity (36-40); morbid obesity (41-50).

- Sedentary lifestyle: defined as the absence of physical activity for at least 10 continuous minutes during the week, categorized as present or absent.

- Comorbidities: evaluation of morbid antecedents, obtained through interviews and classified as: cardiovascular, respiratory, neurological, endocrine, orthopedic, urological, gynecological, psychiatric and rheumatological.

**5.5 Treatments, techniques, tests and exams**

5.5.1 Treatment protocols

For this study, patients will be divided into 2 groups of treatment protocols:

*a) Radiofrequency Group*

The participants will be submitted to three applications, monthly, totaling 12 weeks of treatment. The Wavetronic 6000 Touch device will be used with the Megapulse HF FRAXX system (Loktal Medical Electronics, São Paulo, Brazil), equipped with an electronic energy fractionation circuit, connected to a vaginal pen with 64 microneedles 200µ in diameter and 1mm in length, mounted on a teflon holder and divided into an array of eight columns, with eight needles each. When pressing the trigger pedal, these 64 needles are not energized simultaneously and the energy release is randomized in columns of eight needles in a predefined sequence, which does not allow two adjacent columns to fire in sequence, preventing the thermal sum of the columns (control fractional firing system (Smart Shoot). This allows for cooling between the points and the preservation of tissues adjacent to the vaporized points, so that neocolagenesis and neoelastogenesis can occur, through fibroblastic stimulation. Each shot of the pen performs 64 microablations in the mucosa^68^.

The participant will be placed in the supine position with flexed and abducted lower limbs, in a lithotomy position.

Lidocaine spray 10% will be applied to the vestibule and vaginal opening 3 minutes before the procedure. A disposable vaginal speculum will then be introduced, and thereafter antisepsis with 0.2% aqueous chlorhexidine will be performed, cleaning with 0.9% sterile saline to remove excess vaginal content with gauze. The sequential application of microabactive RF to the vaginal walls under direct vision will be carried out by moving the speculum when necessary. In the vestibule, the application will occur only in the vaginal opening, without including clitoris, foreskin of the clitoris and smaller lips. The electrode will always be kept parallel, lightly touching the mucosa with each shot.

The average procedure time will be 15 to 20 minutes ^68^.

For post-treatment care, the use of 5% dexpanthenol solution in the vaginal opening will be recommended, two to three times a day, for 2 to 5 days, and interruption of sexual intercourse for 10 days ^68^.

All applications will be carried out by the collaborating researcher under the supervision of the principal researchers.

*b) PFMT Group*

The pelvic floor muscle training protocol will be carried out by the collaborating researcher at the Center for Integral Attention to Women's Health - Caism - Hospital da Mulher Dr. José Aristodemo Pinotti. One individual weekly session will be held for 12 weeks, totaling 12 sessions, lasting 60 minutes each, based on the protocol published by Dumoulin *et al.*^69^ and Bo *et al*.^70^. This protocol consists of pelvic mobility, strengthening, resistance and coordination exercises and will be performed in all sessions in different ways, together with pelvic floor contractions, which consist of moderate contractions (3 repetitions / 6 seconds; 3 repetitions / 8 seconds; 3 repetitions / 10 seconds), maximum contractions (6 repetitions / 6 seconds; 8 repetitions / 8 seconds; 10 repetitions / 10 seconds and rapid contractions (2 repetitions / 8 contractions, 2 repetitions / 10 contractions). The exercises will be increased in difficulty, repetition and duration over 12 weeks. Participants will be offered an exercise diary to stimulate home muscle training, following the same scheme of contractions of the pelvic floor muscles.

        For this study, the following equipment will be used:

- Wavetronic 6000 Touch with the Megapulse HF FRAXX system (Loktal Medical Electronics, São Paulo, Brazil), equipped with an electronic energy fractionation circuit, connected to a vaginal pen with 64 microneedles 200µ in diameter and 1mm in length, mounted on a support of teflon and divided into a matrix of eight columns, with eight needles each.

- GE Voluson 730 Expert® transperineal ultrasonography equipment (GE Medical System Kretz-technik GmbH and Co OHG, Zipf, Austria) with 2 to 6 MHz convex RAB4-8L 3D / 4D probe, with the function of recording muscle morphometry the pelvic floor. The probes 4C-D 2 at 5 MHz transabdominal and 5 to 9 MHz transvaginal will be used for vaginal thickness.

5.5.2. Assessment Questionnaires:

Vaginal Laxity Questionnaire (VLQ): a questionnaire containing a question (how do you consider the level of your laxity or vaginal tightening / tightening?) About vaginal laxity with seven ordered responses (very loose = 0, moderately loose = 1, slightly loose = 2, neither loose nor tight = 3, slightly tight = 4, moderately tight = 5, very tight = 6 ^40^.

Marinoff Scale: Depth Dyspareunia Scale (EDP) that evaluates the classification of pain during penetration of the depth of the penis during sexual intercourse. The instrument developed is graded, being zero (absence of pain during sexual intercourse); one (mild pain, which does not force you to interrupt sexual intercourse); two (moderate pain, which makes it difficult, but does not oblige to interrupt sexual intercourse) and three (intense pain, which forces to interrupt sexual intercourse)^61^.

FSFI (Female Sexual Function Index): a brief and multidimensional instrument to assess sexual function in women. The questionnaire was developed and validated by Rosen et al. and consists of 19 items that investigate sexual response over the past four weeks and performance in six domains: sexual desire, arousal, lubrication, orgasm, satisfaction and pain^60^. The validation in Portuguese took place in 2008 by Thiel *et al.* ^71^. The answers are scored according to the sum of the items that make up each domain (simple score) and multiplied by the domain factor generating the weighted score ^71^. The maximum score is 36 points, adding up to the total for each domain. Wiegel et al. proposed a cut-off score to differentiate women with or without sexual dysfunction in the amount of 26.55^72^. Women with scores below the cutoff are classified as sexually dysfunctional. This questionnaire analyzes the sexual response for desire, arousal, vaginal lubrication, orgasm, sexual satisfaction and pain. The total score is the sum of the scores for each domain multiplied by the corresponding factor and can vary from ‘2’ to ‘36’, considering risk for sexual dysfunction a total score less than or equal to ’26 .55 ’.

FSDS-R - Female Sexual Distress Scale-Revised: the scale measures sexual suffering with a composite score greater than or equal to 11, translating to a diagnosis of sexual suffering. Scores less than 11 indicate that there is no distress ^63^. Sexual distress is characterized by a set of feelings (for example, unhappiness, guilt, frustration, stress, worry) and emotions that individuals have about their sexuality. Differs from sexual dysfunction related to symptoms of sexual function, such as arousal, orgasm and pain, separate from emotions ^63^.

ICIQ-SF (International Consultation on Incontinence Questionnaire Short-Form): validated in Portuguese by Tamanini et al., being considered a simple, brief and self-administered questionnaire, capable of quickly and effectively assessing the impact of urinary incontinence on patients' quality of life, in addition to qualifying urinary loss in both sexes. It consists of four questions that assess the frequency, severity and impact of urinary incontinence, in addition to a set of eight self-diagnosis items, related to the causes or situations of urinary incontinence experienced by patients. Your score can vary from 0 to 21, the greater the commitment, the higher the total value ^64^.

ICIQ-VS (International Consultation on Incontinence Questionnaire - Vaginal Symptoms) validated for the Portuguese language by Tamanini *et al.* is a brief questionnaire that assesses the presence and intensity of vaginal symptoms, as well as their relationship with quality of life ^62^.

POP-Q - Pelvic Organ Prolapse Quantification: System frequently used by urogynecologists and the “International Continence Society” (ICS) recommends the description and staging of POP using this instrument ^73, 74^.

The staging classification is defined as ^47^:

Stage 0: There is no demonstrated prolapse.

Stage I: Most of the distal prolapse is more than 1 cm above the level of the hymen.

Stage II: The most distal portion of the prolapse is between 1 cm above the hymen and 1 cm below the hymen.

Stage III: The most distal portion of the prolapse is more than 1 cm beyond the plane of the hymen, but everted at least 2 cm less than the total vaginal length.

Stage IV: Complete eversion or eversion of up to 2 cm from the total length of the lower genital tract.

The hymen is the reference point used to describe the quantitative prolapse and represents the zero point. Patients will be examined in the supine position and measurements taken in centimeters with the aid of a disposable graduated ruler.

Six anatomical points will be evaluated according to ICS guidelines ^47^ (two on the anterior vaginal wall - Aa and Ba, two on the posterior vaginal wall - Ap and Bp and two points on the upper vagina - C and D). The genital hiatus (from the center of the external urethral meatus to the posterior margin of the hymen), the total vaginal length (length of the vagina from the posterior fornix to the hymen will also be measured when point C or D is reduced to its normal position) and the perineal body (from the posterior margin of the hymen to the anal orifice). All points will be measured in maximum Valsalva, except the total vaginal length ^47^.

The ICS has clinically defined the significant POP in stage II or higher ^47, 74^.

5.5.3 Functional and morphometric assessment of the pelvic floor

The patient will be placed in the supine position, with the lower limbs flexed and feet supported on the stretcher. The evaluation of the contractility of the pelvic floor muscles will be performed first through bi-digital palpation, inserting the index and middle fingers 2-3 cm into the vaginal introitus, performing the abduction of the fingers and requesting that the patient perform the maximum voluntary contraction squeezing and cranial movement of the appraiser's fingers. Muscle contractility will be graded according to the modified Oxford scale (0-5)^48^.

The morphometric evaluation protocol of the pelvic floor muscles will consist of measurements at rest, during maximum contraction of the pelvic floor muscles and in the Valsalva maneuver. Before performing the exam, patients will be asked to empty their bladder and be placed in lithotomy with their lower limbs flexed and supported on the stretcher. For the examination, a convex RAB4-8L transducer will be used, covered with water-based gel, protected with a latex protector and positioned longitudinally over the vaginal introitus. Each maneuver will be performed twice and the ultrasound volume with greater angular displacement will be considered for analysis. The evaluated parameters will follow the Cyr protocol *et al.^75^*, with analysis in the mid-sagittal plane and in the axial plane (minimum hiatal dimensions): the position of the bladder neck (positions of the x-axis and the y-axis); the angle of the elevator plate, the anorectal angle, the thickness of the levator ani muscle and the area of the elevator gap in cm2 (anteroposterior measurement and latero-lateral transverse diameters). The ultrasound data will be analyzed offline with the software (4D View, version 10.2; GE Healthcare) by a blinded observer. The vaginal thickness will be assessed in its proximal, middle, and distal third using two approaches - transabdominal and transvaginal^66, 67^.

**5.6 Data collection**

The screening of participants with laxity will be carried out at the Urogynecology Outpatient Clinic by the collaborating researcher. All participants will be guided as to the objectives of the proposal and the evaluation methods by which they will be submitted. Women who accept to participate in the study must sign a Free and Informed Consent Form - FICF (Appendix 10), in two signed copies, one being under the care of the researcher and the other under the care of the volunteer.

Women who accept to participate in the study will be submitted to the evaluation process previously scheduled. At this time, sociodemographic data will be collected, questionnaires applied (appendix 1, 2, 3, 4, 5, 6 and 8), and a functional and morphometric evaluation of the pelvic floor muscles. These procedures will be performed at the gynecology outpatient clinic by the researchers participating in the research.

The 68 women will then be randomized to one of the two research groups. The randomization process will be done through a randomization program by the statistician as the patients are included in the research. The program will select the group that the woman will belong to (isolated RF and isolated PFMT). Each group will be composed of 34 women. Women in the isolated RF group will be instructed to appear once a month for 3 months (totaling 3) in the urogynecology outpatient clinic for RF application. Women in the isolated PFMT group will be invited to attend the physiotherapy sector once a week for 12 weeks. The application of the RF will be made by the collaborating researcher, after previous training with a doctor who has already performed the procedure, in at least 10 cases, to acquire practice in performing the technique.

Women will be instructed to return 30 days and 180 days after the last RF application or the last PFMT session. In these returns, new questionnaires will be applied and new assessments of the pelvic floor function and pelvic organ prolapse will be performed.

In total, women who participate in the PFMT group must attend the service 15 times (for treatment, evaluation and reevaluation) and those who undergo the RF must attend 6 times (for treatment, evaluation and reevaluation).

- 1. **Participants Follow-up**

The study participants, when evaluated, will receive physiotherapeutic guidance relevant to their condition and will be monitored throughout the treatment process and after 30 and 180 days.

- 1. **Criteria for discontinuing, suspending and / or terminating the research**

The discontinuation, suspension and / or termination of the research will occur by checking significant levels of discomfort during application of the vaginal microablative RF, evaluation of the pelvic floor muscles and pelvic organ prolapse, as well as by the significant occurrence of events such as urinary tract infection (UTI), vulvovaginitis and irritation and severe vaginal injury. In these cases, appropriate medical treatment will be offered. Women will be discontinued if they miss any radiofrequency sessions and / or their presence in physiotherapy sessions does not reach 80%.

- 1. **Quality control**

During the data collection stage, the following precautions will be adopted as quality control:

- The randomization with draw and call for treatments will be performed by a single person;

- The questionnaires will be applied by a single researcher, pre and post treatment, he will be blind to what type of therapy was performed;

- The evaluation of the pelvic floor muscles will be performed by a single evaluator experienced in the evaluation of the female pelvic floor muscles, pre and post therapy;

- The application of RF to microablative vaginally will be applied by a single researcher trained to perform it and following the same protocol.

        During the data evaluation stage, the following precautions will be adopted:

- The database will be entered twice by two independent researchers and, if there is a discrepancy between some data, it will be revised;

- The statistician will be blind to groups;

- 1. **Data analysis**

The data analysis will be preceded by the elaboration of a database in the Microsoft Office Excel 2010 application, used for coding the variables in a data dictionary and for validation through double entry (typing) of the data.

Initially, a descriptive analysis of the data will be performed to characterize the research participants, in the form of values of absolute frequency and percentage (relative) for categorical variables and values of mean and standard deviation for numerical variables.

Then, statistical analysis of comparison and correlation of the data obtained from the following statistical tests will be carried out: Kolmogorov-Smirnov to evaluate the normality of the sample, and, depending on the results obtained in the normality test, they will be used for comparative analyzes between groups the Analysis of Variance if the data shows normal distribution or Wilcoxon and Mann-Whitney test if the data are non-parametric Similarly, Pearson or Spearman tests will be used for correlational analyzes. Categorical variables will be analyzed using the chi-square test or Fisher's exact test.

Statistical analyzes will be performed using the statistical program SPSS (Statistical Package for the Social Sciences), adopting a significance level of 5% (p <0.05).

The data will also be evaluated using the method of analysis of variance for repeated measures (ANOVA for repeated measures) with the objective of simultaneously verifying the influence of the 2 study groups (intergroup evaluation) and of the 2 evaluations (intragroup evaluation) for each one. variables, in order to obtain the estimation of the group x time interaction effect. If the numerical variables do not have a normal distribution, they will be transformed into ranks.

- 1. **Dissemination of results**

The results of the study will be made available to research participants and Unicamp. In addition, the results of the research will be sent for publication in jornals of scientific impact and in national and international congresses, with due credits to authors and collaborators.

**6. ETHICAL ASPECTS**

The work will follow the rules of good practice in clinical studies involving human beings, according to Resolution no. 466/12 of the National Health Council, and will be previously approved by the Research Committee of the Center for Integral Attention to Women's Health - CAISM and by the Research Ethics Committee of the State University of Campinas - UNICAMP.

All participants will receive two copies of the Free and Informed Consent Form (Appendix 10) which aims to ensure their rights as a participant, with one copy remaining with the participant and the other with the responsible researcher. At this point, it will be clear to the participant that she may not want to participate or withdraw her authorization at any time, as well as the objectives and methodology of the study will be explained.

Participants will be informed about what the benefits will be as a result of their participation, such as: free evaluation and treatment of vaginal laxity via RF, PFMT or the associated techniques.

They will also be informed about the tests to which they will be submitted as well as the occurrence of discomfort in relation to them. For digital palpation and POP-Q exams, although painless, an anti-allergic lubricating gel will be used to reduce the discomfort caused by the introduction of the examiner's fingers and the graduated ruler.

They will be informed that the application of radiofrequency is a painless procedure, vaginally lasting 15 to 20 minutes. In the vestibule and vaginal opening, 10% lidocaine spray will be applied 3 minutes before the procedure to avoid any discomfort. A disposable vaginal speculum will then be introduced, and thereafter antisepsis will be performed with 0.2% aqueous chlorhexidine, cleaning with 0.9% sterile saline to remove excess vaginal content with gauze.

In addition, the participant will be explained about the guarantee that her identity will be kept confidential and no information will be given to other people who are not part of the research team, emphasizing that in the dissemination of the results of this study, her name will not be mentioned.

**7. BUDGET**

The present study will be carried out on the premises of the State University of Campinas - UNICAMP / SP, since the university already has the necessary infrastructure and equipment for the development of the proposal, such as the physiotherapy team and the electromyography device.

The description of expenses for costing the inputs of the procedures is described in Appendix 10. The rest of the costs will be entirely subsidized by the researchers themselves. The Radiofrequency device is being supplied by the manufacturer Loktal Medical Electronics. It is important to note that the company has no influence on the writing of the project and study design, nor is it remunerating any of the researchers involved directly or indirectly in carrying out this research.

**8. ACTIVITY PLAN AND SCHEDULE**

The study will be carried out over a period of twenty-four months. The research phases are organized in a schedule below.


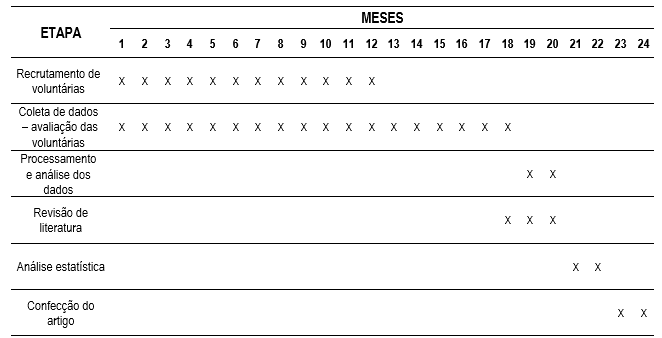
 **Table 1 - Schedule of planned activities.**

**9. REFERENCES**

1. Haylen B, De Ridder D, Freeman R, Swift S, Berghmans B, Lee J. International Continence Society. An International Urogynecological Association (IUGA)/International Continence Society (ICS) joint report on the terminology for female pelvic floor dysfunction. *Neurourol Urodyn*. 2010;29(1):4-20.

2. Singh A, Swift S, Khullar V, Digesu GA. Laser vaginal rejuvenation: not ready for prime time. Springer; 2015.

3. Hamori CA. Aesthetic surgery of the female genitalia: labiaplasty and beyond. *Plastic and reconstructive surgery*. 2014;134(4):661-673.

4. Millheiser LS, Pauls RN, Herbst SJ, Chen BH. Radiofrequency treatment of vaginal laxity after vaginal delivery: nonsurgical vaginal tightening. *The journal of sexual medicine*. 2010;7(9):3088-3095.

5. Pauls RN, Fellner AN, Davila GW. Vaginal laxity: a poorly understood quality of life problem; a survey of physician members of the International Urogynecological Association (IUGA). *International urogynecology journal*. 2012;23(10):1435-1448.

6. Moore R, Miklos J, Chinthakanan O. Evaluation of sexual function outcomes in women undergoing vaginal rejuvenation/vaginoplasty procedures for symptoms of vaginal laxity/decreased vaginal sensation utilizing validated sexual function questionnaire (PISQ-12). *Surgical technology international*. 2014;24:253-260.

7. Dietz HP, Stankiewicz M, Atan IK, Ferreira CW, Socha M. Vaginal laxity: what does this symptom mean? *International urogynecology journal*. 2018:1-6.

8. Berman L, Windecker MA. The relationship between women’s genital self-image and female sexual function: A national survey. *Current Sexual Health Reports*. 2008;5(4):199-207.

9. Dietz HP, Wilson PD, Milsom I. Maternal birth trauma: why should it matter to urogynaecologists? *Current Opinion in Obstetrics and Gynecology*. 2016;28(5):441-448.

10. Sekiguchi Y, Utsugisawa Y, Azekosi Y, et al. Laxity of the vaginal introitus after childbirth: nonsurgical outpatient procedure for vaginal tissue restoration and improved sexual satisfaction using low-energy radiofrequency thermal therapy. *Journal of Women's Health*. 2013;22(9):775-781.

11. Zielinski R, Miller J, Low LK, Sampselle C, DeLancey JO. The relationship between pelvic organ prolapse, genital body image, and sexual health. *Neurourology and urodynamics*. 2012;31(7):1145-1148.

12. Barrett G, Pendry E, Peacock J, Victor C, Thakar R, Manyonda I. Women's sexual health after childbirth. *Bjog*. Feb 2000;107(2):186-95.

13. Griffiths A, Watermeyer S, Sidhu K, Amso N, Nix B. Female genital tract morbidity and sexual function following vaginal delivery or lower segment caesarean section. *Journal of obstetrics and gynaecology*. 2006;26(7):645-649.

14. Aslan E, Fynes M. Female sexual dysfunction. *International Urogynecology Journal*. 2008;19(2):293-305.

15. Yang SH, Yang JM, Wang KH, Huang WC. Biologic correlates of sexual function in women with stress urinary incontinence. *The journal of sexual medicine*. 2008;5(12):2871-2879.

16. Graziottin A, Leiblum SR. Biological and psychosocial pathophysiology of female sexual dysfunction during the menopausal transition. *The Journal of Sexual Medicine*. 2005;2:133-145.

17. Faisal-Cury A, Menezes PR, Quayle J, Matijasevich A, Diniz SG. The relationship between mode of delivery and sexual health outcomes after childbirth. *J Sex Med*. May 2015;12(5):1212-20. doi:10.1111/jsm.12883

18. Klein MC, Gauthier RJ, Robbins JM, et al. Relationship of episiotomy to perineal trauma and morbidity, sexual dysfunction, and pelvic floor relaxation. *Am J Obstet Gynecol*. Sep 1994;171(3):591-8.

19. Signorello LB, Harlow BL, Chekos AK, Repke JT. Postpartum sexual functioning and its relationship to perineal trauma: a retrospective cohort study of primiparous women. *Am J Obstet Gynecol*. Apr 2001;184(5):881-8; discussion 888-90. doi:10.1067/mob.2001.113855

20. Abraham S. Recovery after childbirth. *Med J Aust*. Apr 02 1990;152(7):387.

21. Clarkson J, Newton C, Bick D, et al. Achieving sustainable quality in maternity services - using audit of incontinence and dyspareunia to identify shortfalls in meeting standards. *BMC Pregnancy Childbirth*. 2001;1(1):4.

22. Abdool Z, Shek KL, Dietz HP. The effect of levator avulsion on hiatal dimension and function. *American journal of obstetrics and gynecology*. 2009;201(1):89. e1-89. e5.

23. Dietz HP. PELVIC FLOOR ASSESSMENT. *Fetal and Maternal Medicine Review*. 2009;20(1):49-66.

24. Lien K-C, Mooney B, DeLancey JO, Ashton-Miller JA. Levator ani muscle stretch induced by simulated vaginal birth. *Obstetrics and gynecology*. 2004;103(1):31.

25. Svabik K, Shek K, Dietz H. How much does the levator hiatus have to stretch during childbirth? *BJOG: An International Journal of Obstetrics & Gynaecology*. 2009;116(12):1657-1662.

26. Brooks SV, Zerba E, Faulkner JA. Injury to muscle fibres after single stretches of passive and maximally stimulated muscles in mice. *The Journal of physiology*. 1995;488(2):459-469.

27. Dietz HP, Lanzarone F. Levator trauma after vaginal delivery. *Obstetrics and Gynecology*. Oct 2005;106(4):707-712. doi:10.1097/01.aog.0000178779.62181.01

28. Kearney R, Miller JM, Ashton-Miller JA, DeLancey JO. Obstetric factors associated with levator ani muscle injury after vaginal birth. *Obstet Gynecol*. Jan 2006;107(1):144-9. doi:10.1097/01.AOG.0000194063.63206.1c

29. Dietz HP, Steensma AB. The prevalence of major abnormalities of the levator ani in urogynaecological patients. *Bjog*. Feb 2006;113(2):225-30. doi:10.1111/j.1471-0528.2006.00819.x

30. Shek K, Dietz H. The effect of vaginal childbirth on levator hiatal dimensions. *International Urogynecology Journal*. Sep 2008;19:S130-S130.

31. Shek KL, Dietz HP. Intrapartum risk factors for levator trauma. *Bjog-an International Journal of Obstetrics and Gynaecology*. Nov 2010;117(12):1485-1492. doi:10.1111/j.1471-0528.2010.02704.x

32. Shek KL, Pirpiris A, Dietz HP. Does levator avulsion increase urethral mobility? *Eur J Obstet Gynecol Reprod Biol*. Dec 2010;153(2):215-9. doi:10.1016/j.ejogrb.2010.07.036

33. Jung S-A, Pretorius DH, Padda BS, et al. Vaginal high-pressure zone assessed by dynamic 3-dimensional ultrasound images of the pelvic floor. *American journal of obstetrics and gynecology*. 2007;197(1):52. e1-52. e7.

34. Longcope C. Metabolic clearance and blood production rates of estrogens in postmenopausal women. *American Journal of Obstetrics and Gynecology*. 1971;111(6):778-781.

35. Tan O, Bradshaw K, Carr BR. Management of vulvovaginal atrophy-related sexual dysfunction in postmenopausal women: an up-to-date review. *Menopause*. 2012;19(1):109-117.

36. Cody JD, Jacobs ML, Richardson K, Moehrer B, Hextall A. Oestrogen therapy for urinary incontinence in post‐menopausal women. *Cochrane Database of Systematic Reviews*. 2012;(10)

37. Clobes A, DeLancey JO, Morgan DM. Urethral circular smooth muscle in young and old women. *American journal of obstetrics and gynecology*. 2008;198(5):587. e1-587. e5.

38. Campbell P, Krychman M, Gray T, et al. Self-Reported Vaginal Laxity—Prevalence, Impact, and Associated Symptoms in Women Attending a Urogynecology Clinic. *The journal of sexual medicine*. 2018;15(11):1515-1517.

39. Krychman ML. Vaginal laxity issues, answers and implications for female sexual function. *The journal of sexual medicine*. 2016;13(10):1445-1447.

40. Krychman M, Rowan CG, Allan BB, et al. Effect of single-treatment, surface-cooled radiofrequency therapy on vaginal laxity and female sexual function: the VIVEVE I randomized controlled trial. *The journal of sexual medicine*. 2017;14(2):215-225.

41. Chen L, Ashton-Miller JA, Hsu Y, DeLancey J. Interaction among apical support, levator ani impairment, and anterior vaginal wall prolapse. *Obstetrics and gynecology*. 2006;108(2):324-332.

42. Stein TA, DeLancey JO. Structure of the perineal membrane in females: gross and microscopic anatomy. *Obstetrics and gynecology*. 2008;111(3):686.

43. Corsini-Munt S, Bergeron S, Rosen NO, et al. A comparison of cognitive-behavioral couple therapy and lidocaine in the treatment of provoked vestibulodynia: Study protocol for a randomized clinical trial. Article. *Trials*. 2014;15(1)506. doi:10.1186/1745-6215-15-506.

44. Cundiff GW, Fenner D. Evaluation and treatment of women with rectocele: focus on associated defecatory and sexual dysfunction. *Obstetrics & Gynecology*. 2004;104(6):1403-1421.

45. Lewicky-Gaupp C, Fenner DE, Delancey JO. Posterior vaginal wall repair: Does anatomy matter? *Contemporary Ob/Gyn*. 2009;54(10):44-49.

46. Ghoniem G, Stanford E, Kenton K, et al. Evaluation and outcome measures in the treatment of female urinary stress incontinence: International Urogynecological Association (IUGA) guidelines for research and clinical practice. *International Urogynecology Journal*. 2008;19(1):5-33.

47. Haylen BT, Maher CF, Barber MD, et al. Erratum to: An International Urogynecological Association (IUGA)/International Continence Society (ICS) joint report on the terminology for female pelvic organ prolapse (POP). *International urogynecology journal*. 2016;27(4):655-684.

48. Laycock J. Female pelvic floor assessment: the Laycock ring of continence. *J Natl Women Health Group Aust Physiother Assoc*. 1994:40-51.

49. Practice CoG. ACOG Committee Opinion No. 378: Vaginal" rejuvenation" and cosmetic vaginal procedures. *Obstetrics and gynecology*. 2007;110(3):737.

50. Shafik A. The role of the levator ani muscle in evacuation, sexual performance and pelvic floor disorders. *International Urogynecology Journal*. 2000;11(6):361-376.

51. Bø K, Talseth T, Vinsnes A. Randomized controlled trial on the effect of pelvic floor muscle training on quality of life and sexual problems in genuine stress incontinent women. *Acta obstetricia et gynecologica Scandinavica*. 2000;79(7):598-603.

52. Goldfinger C, Pukall CF, Gentilcore-Saulnier E, McLean L, Chamberlain S. PAIN: A Prospective Study of Pelvic Floor Physical Therapy: Pain and Psychosexual Outcomes in Provoked Vestibulodynia. *The journal of sexual medicine*. 2009;6(7):1955-1968.

53. Dumoulin C, Hay‐Smith J, Habée‐Séguin GM, Mercier J. Pelvic floor muscle training versus no treatment, or inactive control treatments, for urinary incontinence in women: a short version Cochrane systematic review with meta‐analysis. *Neurourology and urodynamics*. 2015;34(4):300-308.

54. Tadir Y, Gaspar A, Lev‐Sagie A, et al. Light and energy based therapeutics for genitourinary syndrome of menopause: consensus and controversies. *Lasers in surgery and medicine*. 2017;49(2):137-159.

55. Elser DM, Mitchell GK, Miklos JR, et al. Nonsurgical transurethral collagen denaturation for stress urinary incontinence in women: 18‐month results from a prospective long‐term study. *Neurourology and urodynamics*. 2010;29(8):1424-1428.

56. Dillon B, Dmochowski R. Radiofrequency for the treatment of stress urinary incontinence in women. *Current urology reports*. 2009;10(5):369-374.

57. Hodgkinson DJ. Clinical applications of radiofrequency: nonsurgical skin tightening (thermage). *Clinics in plastic surgery*. 2009;36(2):261-268.

58. Dunbar SW, Goldberg DJ. Radiofrequency in Cosmetic Dermatology: An Update. *Journal of drugs in dermatology: JDD*. 2015;14(11):1229-1238.

59. Coad J, Vos J, Curtis A, Krychman M. safety And Mechanisms Of Action Supporting Nonablative Radiofrequency Thermal Therapy For Vaginal Introitus Laxity Occurring In Women After Childbirth: Histological Study In The Sheep Vaginal Model: poster# 16. *The Journal of Sexual Medicine*. 2013;10:175.

60. Rosen R, Brown C, Heiman J, et al. The Female Sexual Function Index (FSFI): a multidimensional self-report instrument for the assessment of female sexual function. *J Sex Marital Ther*. Apr-Jun 2000;26(2):191-208. doi:10.1080/009262300278597

61. Mira TA, Giraldo PC, Yela DA, Benetti-Pinto CL. Effectiveness of complementary pain treatment for women with deep endometriosis through Transcutaneous Electrical Nerve Stimulation (TENS): randomized controlled trial. *European Journal of Obstetrics & Gynecology and Reproductive Biology*. 2015;194:1-6.

62. Tamanini JTN, Almeida FG, Girotti ME, Riccetto CL, Palma PC, Rios LAS. The Portuguese validation of the International Consultation on Incontinence Questionnaire—Vaginal Symptoms (ICIQ-VS) for Brazilian women with pelvic organ prolapse. *International Urogynecology Journal*. 2008;19(10):1385-1391.

63. DeRogatis L, Clayton A, Lewis-D'Agostino D, Wunderlich G, Fu Y. Validation of the female sexual distress scale-revised for assessing distress in women with hypoactive sexual desire disorder. *The journal of sexual medicine*. 2008;5(2):357-364.

64. Tamanini JT, Dambros M, D'Ancona CA, Palma PC, Rodrigues Netto N, Jr. [Validation of the "International Consultation on Incontinence Questionnaire -- Short Form" (ICIQ-SF) for Portuguese]. *Rev Saude Publica*. Jun 2004;38(3):438-44. Validacao para o portugues do "International Consultation on Incontinence Questionnaire -- Short Form" (ICIQ-SF). doi:/S0034-89102004000300015

65. Dietz HP. Pelvic floor ultrasound: a review. *American Journal of Obstetrics and Gynecology*. Apr 2010;202(4):321-334. doi:10.1016/j.ajog.2009.08.018

66. Balica A, Wald-Spielman D, Schertz K, Egan S, Bachmann G. Assessing the thickness of the vaginal wall and vaginal mucosa in pre-menopausal versus post-menopausal women by transabdominal ultrasound: A feasibility study. *Maturitas*. Aug 2017;102:69-72. doi:10.1016/j.maturitas.2017.02.017

67. Panayi DC, Digesu GA, Tekkis P, Fernando R, Khullar V. Ultrasound measurement of vaginal wall thickness: a novel and reliable technique. *Int Urogynecol J*. Oct 2010;21(10):1265-70. doi:10.1007/s00192-010-1183-4

68. Kamilos MF, Borrelli CL. New therapeutic option in genitourinary syndrome of menopause: pilot study using microablative fractional radiofrequency. *Einstein (São Paulo)*. 2017;15(4):445-451.

69. Dumoulin C, Morin M, Mayrand MH, Tousignant M, Abrahamowicz M. Group physiotherapy compared to individual physiotherapy to treat urinary incontinence in aging women: study protocol for a randomized controlled trial. *Trials*. Nov 16 2017;18(1):544. doi:10.1186/s13063-017-2261-4

70. Bø K, Talseth T, Holme I. Single blind, randomised controlled trial of pelvic floor exercises, electrical stimulation, vaginal cones, and no treatment in management of genuine stress incontinence in women. *Bmj*. Feb 20 1999;318(7182):487-93. doi:10.1136/bmj.318.7182.487

71. Thiel Rdo R, Dambros M, Palma PC, Thiel M, Riccetto CL, Ramos Mde F. [Translation into Portuguese, cross-national adaptation and validation of the Female Sexual Function Index]. *Rev Bras Ginecol Obstet*. Oct 2008;30(10):504-10. Traducao para portugues, adaptacao cultural e validacao do Female Sexual Function Index.

72. Wiegel M, Meston C, Rosen R. The Female Sexual Function Index (FSFI): Cross-validation and development of clinical cutoff scores. *Journal of Sex & Marital Therapy*. Jan-Feb 2005;31(1):1-20. doi:10.1080/00926230590475206

73. Bump RC, Mattiasson A, Bø K, et al. The standardization of terminology of female pelvic organ prolapse and pelvic floor dysfunction. *American journal of obstetrics and gynecology*. 1996;175(1):10-17.

74. Garnham AP, Rojas RG, Shek KL, Dietz HP. Predicting levator avulsion from ICS POP-Q findings. *International Urogynecology Journal*. 2014:1-5.

75. Cyr MP, Kruger J, Wong V, Dumoulin C, Girard I, Morin M. Pelvic floor morphometry and function in women with and without puborectalis avulsion in the early postpartum period. *Am J Obstet Gynecol*. Mar 2017;216(3):274.e1-274.e8. doi:10.1016/j.ajog.2016.11.1049

76. Ferreira CHJ, Barbosa PB, de Oliveira Souza F, Antônio FI, Franco MM, Bø K. Inter-rater reliability study of the modified Oxford Grading Scale and the Peritron manometer. *Physiotherapy*. 2011;97(2):132-138.

1. **RESEARCH DISSEMINATION**

The research will be published on social media, in newspapers, on radio and on television programs with the aim of assisting in the process of recruiting volunteer patients, by reading the text, reporting or disseminating the printed text or in postings on social media.

VAGINAL LAXITY RESEARCH

The State University of Campinas - UNICAMP invites women over 18 years old, with at least one normal delivery (vaginal) and complaining of vaginal laxity (wide vagina) to participate in the Vaginal Looseness Survey. The research will be carried out at Caism - Hospital da Mulher J. A. Pinotti, with the support of the Urogynecology team and aims to evaluate two free treatment options - physiotherapy and radiofrequency.

Physiotherapy plays an important role in female sexual function by strengthening the muscles that support the vagina.

Radiofrequency is an innovative treatment that acts on the vaginal layers providing greater resistance to the vagina. Radiofrequency uses high technology through vaginal and painless treatment.

Ask questions and get informed by phone (WhatsApp) 19 98176 7113 or by email: researchfrouxidaovaginal@gmail.com

Be alert:

Vaginal laxity or wide vagina is defined as excess vaginal flaccidity, occurring at any age and can affect women's sexual lives and relationships.

**APPENDIX 1 – Vaginal Laxity Questionnaire (VLQ)**

How would you rate your current level of vaginal laxity? or laxity during intercourse? SCORE ______

1- Very loose; 2- Moderately loose; 3- Slightly loose; 4- Neither loose nor tight; 5- Slightly tight; 6- Moderately tight; 7- Too tight

**APPENDIX 2 - Female Sexual Function Index (FSFI)**

**Female Sexual Function Index (FSFI)**

These questions are about your sexual feelings and responses over the past FOUR WEEKS. Please answer the following questions as clearly and honestly as possible. Your answers will be kept completely confidential. The following definitions (explanations) are applied to answer the questionnaire: Sexual activity: may include caresses, preliminary sexual stimulation, masturbation and vaginal intercourse. Sexual intercourse is defined as the penetration (entry) of the penis into the vagina. Sexual stimulation: includes preliminary sexual stimulation with the partner, autoeroticism (masturbation) or sexual fantasy.

FOR EACH ITEM, JUST ONE ANSWER ONLY

Sexual desire or interest is a feeling that encompasses the desire to have a sexual experience, the receptivity to the partner's sexual initiatives, and thoughts or fantasies about the sexual act.

1. Over the past 4 weeks, how often have you felt sexual desire or interest?

(5) Always or almost always

(4) Often (more than half the time)

(3) Sometimes (approximately half the time)

(2) Few times (less than half the time)

(1) Never or almost never

2. Over the past 4 weeks, how would you rate your level (degree) of sexual desire

or interest?

(5) Very high

(4) High

(3) Moderate

(2) Low

(1) Very low or none

Sexual arousal is a feeling that includes both physical and mental aspects of sexual

excitement. It may include feelings of warmth or tingling in the genitals, lubrication

(wetness), or muscle contractions.

3. Over the past 4 weeks, how often did you feel sexually aroused ("turned on") during sexual activity or intercourse?

(0) No sexual activity

(5) Always or almost always

(4) Often (more than half the time)

(3) Sometimes (half the time)

(2) Few times (less than half the time)

(1) Never or almost never

4. Over the past 4 weeks, how would you rate your level of sexual arousal ("turn on") during sexual activity or intercourse?

(0) No sexual activity

(5) Very high

(4) High

(3) Moderate

(2) Low

(1) Very low or none

5. Over the past 4 weeks, how confident were you about becoming sexually aroused during sexual activity or intercourse?

(0) No sexual activity

(5) Highest confidence

(4) High confidence

(3) Moderate confidence

(2) Low confidence

(1) Very low or no confidence

6. Over the past 4 weeks, how often have you been satisfied with your arousal (excitement) during sexual activity or intercourse?

(0) No sexual activity

(5) Always or almost always

(4) Often (more than half the time)

(3) Sometimes (approximately half the time)

(2) Few times (less than half the time)

(1) Never or almost never

7. Over the past 4 weeks, how often did you become lubricated ("wet") during sexual activity or intercourse?

(0) No sexual activity

(5) Always or almost always

(4) Often (more than half the time)

(3) Sometimes (approximately half the time)

(2) Few times (less than half the time)

(1) Never or almost never

8. Over the past 4 weeks, how difficult was it to become lubricated ("wet") during sexual activity or intercourse?

(0) No sexual activity

(1) Extremely difficult or impossible

(2) Very difficult

(3) Difficult

(4) Little difficult

(5) Not difficult

9. Over the past 4 weeks, how often did you maintain your lubrication ("wetness") until completion of sexual activity or intercourse?

(0) No sexual activity

(5) Always or almost always

(4) Often (more than half the time)

(3) Sometimes (approximately half the time)

(2) Few times (less than half the time)

(1) Never or almost never

10. Over the past 4 weeks, how difficult was it to maintain your lubrication ("wetness") until completion of sexual activity or intercourse?

(0) No sexual activity

(1) Extremely difficult or impossible

(2) Very difficult

(3) Difficult

(4) Little Difficult

(5) Not Difficult

11. Over the past 4 weeks, when you had sexual stimulation or intercourse, how

often did you reach orgasm (climax)?

(0) No sexual activity

(5) Always or almost always

(4) Often (more than half the time)

(3) Sometimes (approximately half the time)

(2) Few times (less than half the time)

(1) Never or almost never

12. Over the past 4 weeks, when you had sexual stimulation or intercourse, how

difficult was it for you to reach orgasm (climax)?

(0) No sexual activity

(1) Extremely difficult or impossible

(2) Very difficult

(3) Difficult

(4) Little Difficult

(5) Not Difficult

13. Over the past 4 weeks, how satisfied were you with your ability to reach orgasm

(climax) during sexual activity or intercourse?

(0) No sexual activity

(5) Very satisfied

(4) Moderately satisfied

(3) Indifferent

(2) Moderately dissatisfied

(1) Very dissatisfied

14. Over the past 4 weeks, how satisfied have you been with the amount of

emotional closeness during sexual activity between you and your partner?

(0) No sexual activity

(5) Very satisfied

(4) Moderately satisfied

(3) Indifferent

(2) Moderately dissatisfied

(1) Very dissatisfied

15. Over the past 4 weeks, how satisfied have you been with your sexual

relationship with your partner?

(5) Very satisfied

(4) Moderately satisfied

(3) Indifferent

(2) Moderately dissatisfied

(1) Very dissatisfied

16. Over the past 4 weeks, how satisfied have you been with your overall sexual life?

(5) Very satisfied

(4) Moderately satisfied

(3) Indifferent

(2) Moderately dissatisfied

(1) Very dissatisfied

17. Over the past 4 weeks, how often did you experience discomfort or pain during

vaginal penetration?

(0) There was no attempt to penetrate

(1) Always or almost always

(2) Often (more than half the time)

(3) Sometimes (approximately half the time)

(4) Few times (less than half the time)

(5) Never or almost never

18. Over the past 4 weeks, how often did you experience discomfort or pain following

vaginal penetration?

(0) There was no attempt to penetrate

(1) Always or almost always

(2) Often (more than half the time)

(3) Sometimes (approximately half the time)

(4) Few times (less than half the time)

(5) Never or almost never

19. Over the past 4 weeks, how would you rate your level (degree) of discomfort or

pain during or following vaginal penetration?

(0) There was no attempt to penetrate

(1) Very high

(2) High

(3) Moderate

(4) Low

(5) Very low or none

**FSFI DOMAIN SCORES AND FULL SCALE SCORE**
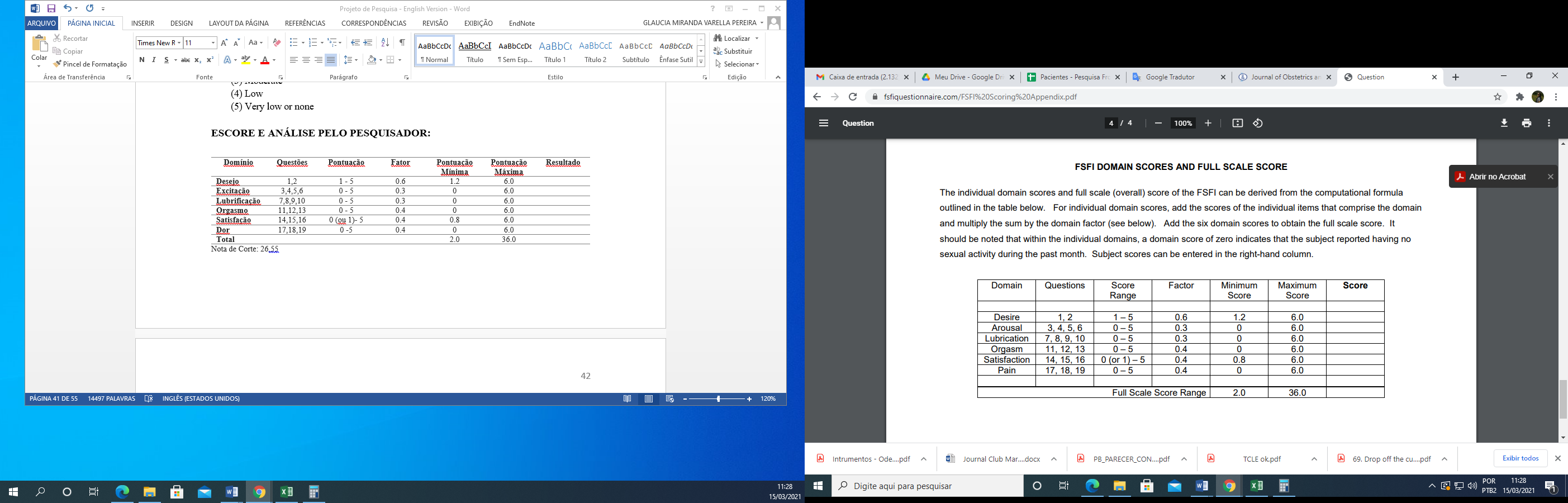


Cut-off: 26,55

**APPENDIX 3- Depth Dyspareunia Scale, based on the Marinoff Scale**

About pain in intercourse:

| No sexual intercourse |  | No |
| --- | --- | --- |
| No pain with intercourse |  | 0 |
| Pain with intercourse that doesn’t prevent the completion |  | 1 |
| Pain with intercourse requiring interruption or discontinuance |  | 2 |
| Pain with intercourse preventing any intercourse |  | 3 |

**APPENDIX 4 - International Consultation on Incontinence Questionnaire - Vaginal Symptoms (ICIQ-VS)**

1.    Are you aware of dragging pain in your lower abdomen?

| Never |  | 0 |
| --- | --- | --- |
| Occasionally |  | 1 |
| Sometimes |  | 2 |
| Mosto of the time |  | 3 |
| All the time |  | 4 |

How much does this bother you? Please ring a number between 0 (not at all) and 10 (a great deal) 0       1       2        3 4       5        6 7       8        9 10

2.   Are you aware of soreness in your vagina?

| Never |  | 0 |
| --- | --- | --- |
| Occasionally |  | 1 |
| Sometimes |  | 2 |
| Mosto of the time |  | 3 |
| All the time |  | 4 |

How much does this bother you? Please ring a number between 0 (not at all) and 10 (a great deal) 0       1       2        3 4       5        6 7       8        9 10

3.    Do you feel that you have reduced sensation or feeling in or around your vagina?

| Not at all |  | 0 |
| --- | --- | --- |
| A little |  | 1 |
| Somewhat |  | 2 |
| A lot |  | 3 |

How much does this bother you? Please ring a number between 0 (not at all) and 10 (a great deal) 0       1       2        3 4       5        6 7       8        9 10

4.    Do you feel that your vagina is too loose or lax?

| Not at all |  | 0 |
| --- | --- | --- |
| A little |  | 1 |
| Somewhat |  | 2 |
| A lot |  | 3 |

How much does this bother you? Please ring a number between 0 (not at all) and 10 (a great deal) 0       1       2        3 4       5        6 7       8        9 10

5.    Are you aware of a lump or bulge coming down in your vagina?

| Never |  | 0 |
| --- | --- | --- |
| Occasionally |  | 1 |
| Sometimes |  | 2 |
| Mosto of the time |  | 3 |
| All the time |  | 4 |

How much does this bother you? Please ring a number between 0 (not at all) and 10 (a great deal) 0       1       2        3 4       5        6 7       8        9 10

6.    Do you feel a lump or bulge come out of your vagina, so that you can feel it on the outside or see it on the outside?

| Never |  | 0 |
| --- | --- | --- |
| Occasionally |  | 1 |
| Sometimes |  | 2 |
| Mosto of the time |  | 3 |
| All the time |  | 4 |

How much does this bother you? Please ring a number between 0 (not at all) and 10 (a great deal) 0       1       2        3 4       5        6 7       8        9 10

7. Do you feel that your vagina is too dry?

| Never |  | 0 |
| --- | --- | --- |
| Occasionally |  | 1 |
| Sometimes |  | 2 |
| Mosto of the time |  | 3 |
| All the time |  | 4 |

How much does this bother you? Please ring a number between 0 (not at all) and 10 (a great deal) 0       1       2        3 4       5        6 7       8        9 10

8. Do you have to insert a finger into your vagina to help empty your bowels?

| Never |  | 0 |
| --- | --- | --- |
| Occasionally |  | 1 |
| Sometimes |  | 2 |
| Mosto of the time |  | 3 |
| All the time |  | 4 |

How much does this bother you? Please ring a number between 0 (not at all) and 10 (a great deal) 0       1       2        3 4       5        6 7       8        9 10

9. Do you feel that your vagina is too tight?

| Never |  | 0 |
| --- | --- | --- |
| Occasionally |  | 1 |
| Sometimes |  | 2 |
| Mosto of the time |  | 3 |
| All the time |  | 4 |

How much does this bother you? Please ring a number between 0 (not at all) and 10 (a great deal) 0       1       2        3 4       5        6 7       8        9 10

10. Do you have a sex life at present?

Yes - 1

no, because of my vaginal symptoms - 0

no, because of other reasons - 2

11.Do worries about your vagina interfere with your sex life?

| Not at all |  | 0 |
| --- | --- | --- |
| A little |  | 1 |
| Somewhat |  | 2 |
| A lot |  | 3 |

How much does this bother you? Please ring a number between 0 (not at all) and 10 (a great deal) 0       1       2        3 4       5        6 7       8        9 10

12**.** Do you feel that your relationship with your partner is affected by vaginal symptoms?

| Not at all |  | 0 |
| --- | --- | --- |
| A little |  | 1 |
| Somewhat |  | 2 |
| A lot |  | 3 |

How much does this bother you? Please ring a number between 0 (not at all) and 10 (a great deal) 0       1       2        3 4       5        6 7       8        9 10

13**.** How much do you feel that your sex life has been spoilt by vaginal symptoms?

Please ring a number between 0 (not at all) and 10 (a great deal)

0       1       2        3 4       5        6 7       8        9 10

**APPENDIX 5 – The Female Sexual Distress Scale-Revised *–* (FSDSR)**

0—never, 1—rarely, 2—occasionally, 3—frequently, 4—always.

Questions (how often did you feel—)

1. Distressed about your sex life? ( 0 ) ( 1 ) ( 2 ) ( 3 ) ( 4 )

2. Unhappy about your sexual relationship? ( 0 ) ( 1 ) ( 2 ) ( 3 ) ( 4 )

3. Guilty about sexual difficulties? ( 0 ) ( 1 ) ( 2 ) ( 3 ) ( 4 )

4. Frustrated by your sexual problem? ( 0 ) ( 1 ) ( 2 ) ( 3 ) ( 4 )

5. Stressed about sex? ( 0 ) ( 1 ) ( 2 ) ( 3 ) ( 4 )

6. Inferior because of sexual problems? ( 0 ) ( 1 ) ( 2 ) ( 3 ) ( 4 )

7. Worried about sex? ( 0 ) ( 1 ) ( 2 ) ( 3 ) ( 4 )

8. Sexually inadequate? ( 0 ) ( 1 ) ( 2 ) ( 3 ) ( 4 )

9. Regrets about your sexuality? ( 0 ) ( 1 ) ( 2 ) ( 3 ) ( 4 )

10. Embarrassed about sexual problems? ( 0 ) ( 1 ) ( 2 ) ( 3 ) ( 4 )

11. Dissatisfied with your sexual life? ( 0 ) ( 1 ) ( 2 ) ( 3 ) ( 4 )

12. Angry about your sexual life? ( 0 ) ( 1 ) ( 2 ) ( 3 ) ( 4 )

13. Bothered by your partner’s premature ejaculation? ( 0 ) ( 1 ) ( 2 ) ( 3 ) ( 4 )

SCORE:

**APPENDIX 6 - International Consultation on Incontinence Questionnaire Urinary Incontinence – Short Form (ICIQ UI-SF)**

- How often do you leak urine?

| *Never* |  | *0* |
| --- | --- | --- |
| About once a week or less often |  | *1* |
| Two or three times a week |  | *2* |
| About once a day |  | *3* |
| Several times a day |  | *4* |
| All the time |  | *5* |

- How much urine do you usually leak?

| None |  | 0 |
| --- | --- | --- |
| Small amount |  | 2 |
| A moderate amount |  | 4 |
| A large amount |  | 6 |

Overall, how much does leaking urine interfere with your everyday life? Please ring a number between 0 (not at all) and 10 (a great deal).

0 1 2 3 4 5 6 7 8 9 10

**ICIQ-SF Score (1+2+3) = _______________**

- When does urine leak?

| Never |  | 0 |
| --- | --- | --- |
| Leaks before you can get to the toilet |  | 1 |
| Leaks when you cough or sneeze |  | 2 |
| Leaks when you are asleep |  | 3 |
| Leaks when you are physically active/exercising |  | 4 |
| Leaks when you have finished urinating and are dressed |  | 5 |
| Leaks for no obvious reason |  | 6 |
| Leaks all the time |  | 7 |

Note: Variable score from 0 to 21 points. The higher the score, the greater the commitment.

**APPENDIX 7 – Pelvic Organ Prolapse Quantification** ***–* (POP-Q)**

Stage 0: There is no demonstrated prolapse.

Stage I: Most of the distal prolapse is more than 1 cm above the level of the hymen.

Stage II: The most distal portion of the prolapse is between 1 cm above the hymen and 1 cm below the hymen.

Stage III: The most distal portion of the prolapse is more than 1 cm beyond the plane of the hymen, but everted at least 2 cm less than the total vaginal length.

Stage IV: Complete eversion or eversion of up to 2 cm from the total length of the tract

| **PROLAPSE EVALUATION: STAGING: ________________** | | | | | | |  |  |  |
| --- | --- | --- | --- | --- | --- | --- | --- | --- | --- |
| Aa (+3 -3) _________ |  |  | Ba (+3 -3) _________ |  |  | C __________ cm | |  |  |
| Gh ____________cm | |  | Bp _____________cm | |  | TVL _________cm | |  |  |
| Ap (+3 -3)_________ |  |  | Bp (+3 -3)_________ |  |  | D ____________cm | |  |  |
| **URETHAL HYPERMERBILITY:**  □ YES □ NO | | | | | | | | | |

Aa and Ba = anterior vaginal wall

Ap and Bp = posterior vaginal wall

C and D = upper vagina

GH = genital hiatus (from the center of the external urethral meatus to the posterior margin of the hymen

TVL = total vaginal length (length of the vagina from the posterior fornix to the hymen when point C or D is reduced to its normal position

PB = perineal body (from the posterior margin of the hymen to the anal orifice)

**APPENDIX 8 - Modified Oxford Scale and Ultrasonography**

Oxford scale modified by Laycock ^48^:

0: without objective perineal function;

1: outline of muscle contraction;

2: weak intensity contraction;

3: contraction of regular intensity and cranial elevation of the vaginal wall;

4: good intensity contraction and cranial elevation of the vaginal wall;

5: contraction of optimal intensity and cranial elevation of the vaginal wall;

SCORE: _______________

Morphometry Protocol - Ultrasonography ^65, 75^

Measure at rest:______________________________________________________

**_____________________________________________________________________________**

Measure at Valsalva: ___________________________________________________________

_____________________________________________________________________________

Measure at contraction: _________________________________________________________

_____________________________________________________________________________

Vaginal Thickness:

Abdominal:_________________________________________________________________

Transvaginal: _______________________________________________________________

**APPENDIX 9 - Free and Informed Consent Term (FICT)**

**FREE AND CLARIFIED CONSENT TERM**

**Effect of Fractionated Microablative Radiofrequency and Pelvic Floor Muscle Training in the Treatment of Women with Vaginal Laxity Complaint: Randomized Clinical Trial**

Gláucia Miranda Varella Pereira; Cássia Raquel Teatin Juliato**;** Luiz Gustavo Oliveira Brito;

**Número do CAAE:** 12919119.9.0000.5404

You are being invited to participate in a research. This document, called the Free and Informed Consent Form, aims to ensure your rights as a research participant and is prepared in two copies, signed and initialed by the researcher and the participant / legal guardian, one copy of which must remain with you and the other with the researcher.

Please read carefully and calmly, taking the opportunity to clarify your doubts. If there are questions before or even after signing it, you can clarify them with the researcher. If you prefer, you can take this Term home and consult your family or others before deciding to participate. There will be no penalty or loss if you do not agree to participate or withdraw your authorization at any time. If you withdraw your authorization or do not want to participate, you will be referred to the conventional treatment present in the service.

**Justification and objectives:**

You are being invited to participate in a study that seeks to evaluate two types of treatment for your complaint of vaginal laxity. A treatment option will be physiotherapy, through muscle training of the pelvic floor, the benefit of which is to strengthen the muscles of the vagina region. The other type of treatment is called radiofrequency - it is a device that will be placed in the region of the vagina, whose goal is to improve vaginal elasticity. For this, you will be selected for a treatment group and will remain in that group until the end of the treatment proposal. We do not know which of these treatments is best for treating your complaint, so we are conducting this research.

**Procedures:**

Participating in the study you will be invited to:

- answer some general questions such as your age, weight, number of deliveries, etc.

_________________________ _____________________________ Page 1 de 4

Researcher rubric Participant rubric

- answer questionnaires about urine loss, vaginal prolapse, symptoms in the vagina and sexual activity and satisfaction, which will take an average of 15 minutes in total.

-be subjected to physical examinations to assess the muscles that support the vagina (vaginal route - vaginal touch and vaginal probe), examination to evaluate vaginal prolapse (vaginal route with graduated ruler) and ultrasound on the perineum (without introducing it into the vagina). Physical exams will take an average of 30 - 40 minutes.

The questionnaires and all parameters will be carried out before treatment and 30 days and 6 months after the end of treatment. A raffle will define which treatment you will receive: isolated radio frequency or isolated physiotherapy. You will not be able to choose which treatment to perform. If a radiofrequency procedure is performed, there will be 3 monthly, painless and intravaginal applications. If it is physiotherapy, group sessions will be held once a week for 12 weeks. You will also carry out the treatment at home and will receive instructions for carrying it out.

**Discomfort and risk:**

You may feel uncomfortable answering questions and being examined gynecologically. No discomfort is expected during or after physical therapy. Radiofrequency is a painless procedure, but it can cause mild discomfort in some patients during its application. Radiofrequency has no side effect such as secretion, bleeding, chronic pain, infection or cancer. You should not have sexual intercourse 3 days before the radio frequency, nor should you use intravaginal ointments or creams. After each radio frequency session, you will not be able to have sex for 10 days.

**Benefits:**

You will benefit from access to specialized treatment for vaginal laxity, with radiofrequency or physiotherapy, and in return will contribute to a better understanding of the treatments for vaginal laxity. If the results of the research show that one group is better than the other for treatment, if you are in the group that has not shown this benefit, you will have the right to treat the other group after the end of the research, if you wish.

**Monitoring and assistance:**

If there is any problem that does not allow you to participate in the study, you will be referred to the outpatient clinic for surgical gynecology and or to the physical therapy sector of CAISM, even if you no longer wish to participate in the research. The research will not change your treatment in any way if you decide not to participate in the research. You will have your follow-up guaranteed, even after the end of the survey, to be evaluated in case of complaints that may be related to the survey, regardless of the end of the survey. In case of missed appointments previously scheduled for exams or radiofrequency or physical therapy procedures without justification, you will be disconnected from the research and another volunteer will be invited**.**

__________________________ _____________________________ Page 2 de 4

Research Rubric Participant Rubric

**Secrecy and privacy:**

You have the guarantee that your identity will be kept confidential and no information will be given to other people who are not part of the research team. When disclosing the results of this study, your name will not be mentioned. The results of this research will not be in your medical record.

**Reimbursement and Indemnity:**

You will not receive any living allowance to participate in the survey. The research will be carried out during the physiotherapy sessions that you would have scheduled (treatment routine defined by the service). This way, you will not have extra expenses to participate in the research. You will be entitled to compensation in cases of direct and indirect damages resulting from the research. There will be no cost to perform the exams and neither will the radiofrequency treatment (if this is the chosen treatment). All exams will be performed on the same day of the assessment or physiotherapeutic treatment in order to facilitate your displacement.

**Contact:**

In case of questions about the research, you can contact the researchers Gláucia Varella or Luiz Gustavo Brito: Rua Alexandre Fleming, 79 Campinas - SP; phone (WhatsApp) (19) 9 8176 7113.

In case of denunciations or complaints about your participation and about ethical issues of the study, you can contact the secretariat of the Research Ethics Committee (CEP) of UNICAMP from 8:00 am to 11:30 am and from 1:00 pm to 5:30 pm on the street: Tessália Vieira de Camargo, 126; CEP 13083-887 Campinas - SP; telephone (19) 3521-8936 or (19) 3521-7187; e-mail: cep@fcm.unicamp.br.

**The Research Ethics Committee (REC).**

REC's role is to evaluate and monitor the ethical aspects of all research involving human beings. The National Commission for Ethics in Research (CONEP), aims to develop regulations on the protection of human beings involved in research. It plays a coordinating role in the network of Research Ethics Committees (RECs) of the institutions, in addition to assuming the role of an advisory body in the area of research ethics.

__________________________ _____________________________ Page 3 de 4

Research Rubric Participant Rubric

**Informed consent:**

After receiving clarifications on the nature of the research, its objectives, methods, expected benefits, potential risks and the inconvenience that this may cause, I accept to participate:

Name of the research participant: ________________________________________

_________________________________________________Date: ____/_____/______

(Signature of the research participant or name and signature of his / her LEGAL RESPONSIBLE)

**Researcher Responsibility:**

I assure you that I have complied with the requirements of resolution 466/2012 CNS / MS and complementary in the elaboration of the protocol and in obtaining this Informed Consent Form. I also assure you that I have explained and provided a copy of this document to the research participant. I inform you that the study was approved by the CEP before which the project was presented and by CONEP, when pertinent. I undertake to use the material and data obtained in this research exclusively for the purposes set out in this document or according to the consent given by the research participant.

_________________________________________________Date: ____/_____/______.

(Researcher's signature)

Page 4 de 4

**APPENDIX 10 -Budget**

| **UNIVERSIDADE ESTADUAL DE CAMPINAS**  **HOSPITAL DA MULHER PROF. DR. JOSÉ ARISTODEMO PINOTTI – CAISM** | | | | | |
| --- | --- | --- | --- | --- | --- |
| **BUDGET - ESTIMATE** | | | | | |
| **TITLE**: **Effect of Fractionated Microablative Radiofrequency and Pelvic Floor Muscle Training in the Treatment of Women with Vaginal Laxity Complaint: Randomized Clinical Trial**  **PRINCIPAL RESEARCHER:** Dr. Luiz Gustavo Oliveira Brito  **COLLABORATING RESEARCHER:** Gláucia Miranda Varella Pereira | | | | | |
| **Materials** | | | | | |
| Item | Description | Unit | Amount | Value Unit | Total Value |
| 1 | Speculum | unit | 315 | R$ 1,87 | R$589,05 |
| 2 | Lidocaine Spray 50mL | unit | 10 | R$68,00 | R$680,00 |
| 3 | Chlorhexidine 100mL | unit | 20 | R$ 4,00 | R$ 80,00 |
| 4 | Sterile Saline Solution | unit | 55 | R$ 5,00 | R$ 275,00 |
| 5 | Gauze | unit | 100 | R$ 1,10 | R$ 110,00 |
| 6 | Gel | Kg | 5 | R$11,00 | R$ 55,00 |
| 7 | Tongue depressor | Package | 3 | R$ 5,98 | R$ 17,94 |
| **Permanent Item: Not applicable** | | | | | |
| **Third Party Service: Not applicable** | | | | | |
| **Rates: Not applicable** | | | | | |
| **Transport Expenses: Not applicable** | | | | | |
| **Total Expenses:** R$ 1.806,99 | | | | | |
