# Supplementary material for: Effect of radiofrequency and pelvic floor muscle training in the treatment of women with vaginal laxity: A study protocol
Source: PLoS One. 2021 Nov 9;16(11):e0259650. doi: 10.1371/journal.pone.0259650 (PMC8577744; doi:10.1371/journal.pone.0259650)
Supplement: S2 File — (DOCX) [file pone.0259650.s003.docx]

___________________________________________________________

**UNIVERSIDADE ESTADUAL DE CAMPINAS**

**Faculdade de Ciências Médicas**

**Efeito da Radiofrequência Microablativa Fracionada e do Treinamento dos Músculos do Assoalho Pélvico no Tratamento de Mulheres com Queixa de Frouxidão Vaginal: Ensaio Clínico Randomizado**

**PROJETO DE PESQUISA - DOUTORADO**

**PESQUISADORES RESPONSÁVEIS: Prof. Dr. Luiz Gustavo Oliveira Brito**

**Profa. Dra. Cássia Raquel Teatin Juliato**

**PESQUISADORA COLABORADORA: Gláucia Miranda Varella Pereira**

**Autorizo a realização desta pesquisa:**

_______________________________________

*Profa. Dra. Lucia Helena S. Costa Paiva*

Divisão de Ginecologia do Centro de Atenção à Saúde Integral da Mulher – CAISM/UNICAMP

**Campinas**

**10/04/2019**

**IDENTIFICAÇÃO DO PROJETO**

**Título:** Efeito da radiofrequência microablativa fracionada e do treinamento dos músculos do assoalho pélvico no tratamento de mulheres com queixa de frouxidão vaginal: ensaio clínico randomizado

**Instituição executora/proponente:** Universidade Estadual de Campinas – UNICAMP - Faculdade de Ciências Médicas – Departamento de Tocoginecologia – Divisão de Ginecologia

Local de desenvolvimento da proposta: Ambulatório de Cirurgia Ginecológica do Centro de Atenção Integral à Saúde da Mulher – CAISM da Universidade Estadual de Campinas – UNICAMP.

**IDENTIFICAÇÃO DOS PESQUISADORES**

**Pesquisador principal:** Prof. Dr. Luiz Gustavo Oliveira Brito

**Cargo/Função:** Docente do Departamento de Tocoginecologia da Faculdade de Ciências Médicas – UNICAMP/SP.

**RG:** 79.972.797 - 0

**CPF:** 833. 347. 693 – 72

**Endereço:** Universidade Estadual de Campinas, Faculdade de Ciências Médicas, Departamento de Tocoginecologia – Centro de Atenção Integral à saúde da Mulher (CAISM), Rua Tessália Vieira de Camargo, 126. Cidade Universitária “Zeferino Vaz”. CEP-13083-887 – Campinas, SP – Brasil.

**E-mail:** [lgobrito@gmail.com](mailto:lgobrito@gmail.com) / [lgobrito@unicamp.br](mailto:lgobrito@unicamp.br)

**Telefone para contato:** 19-3521-9595

**Currículo plataforma *Lattes*:** <http://lattes.cnpq.br/0402956055809744>

**Assinatura:**____________________________________________________________

**Pesquisadora colaboradora:** Prof. Dra. Cássia Raquel Teatin Juliato

**Cargo/Função:** Médica ginecologista, Docente do Departamento de Tocoginecologia da Faculdade de Ciências Médicas – UNICAMP/SP.

**RG:** 25. 311. 099 - 3

**CPF:** 255. 104. 518 - 58

**Endereço:** Universidade Estadual de Campinas, Faculdade de Ciências Médicas, Departamento de Tocoginecologia – Centro de Atenção Integral à saúde da Mulher (CAISM), Rua Tessália Vieira de Camargo, 126. Cidade Universitária “Zeferino Vaz”. CEP-13083-887 – Campinas, SP – Brasil.

**E-mail:** cassia.raquel@gmail.com      **Telefone para contato:** (19) 35219516

**Currículo plataforma *Lattes*:** <http://lattes.cnpq.br/2246374748063899>

**Assinatura:**____________________________________________________________

**Pesquisadora colaboradora:** Gláucia Miranda Varella Pereira

**Cargo/Função:** Fisioterapeuta mestre em Saúde da Mulher e aluna de doutorado da Pós-Graduação em Tocoginecologia da Universidade Estadual de Campinas – UNICAMP/SP.

**RG:** 12.232.338

**CPF:** 046.330.266 -40

**Endereço:** Universidade Estadual de Campinas, Faculdade de Ciências Médicas, Departamento de Tocoginecologia – Centro de Atenção Integral à saúde da Mulher (CAISM), Rua Tessália Vieira de Camargo, 126. Cidade Universitária “Zeferino Vaz”. CEP-13083-887 – Campinas, SP – Brasil.

**E-mail:** glauciavarella@gmail.com **Telefone para contato:** (37)99997 7771

**Currículo plataforma *Lattes*:** [http://lattes.cnpq.br/8072128386816527](https://wwws.cnpq.br/cvlattesweb/PKG_MENU.menu?f_cod=347C552F5472035B101ACB6F3A5A3BD9)

**Assinatura:**____________________________________________________________

**RESUMO**

**Introdução:** A frouxidão vaginal, condição raramente discutida entre as pacientes e médicos, é definida como queixa de excesso de flacidez vaginal. Apresenta prevalência de 24% e parece estar associada à idade jovem, partos vaginais, sintomas de prolapso e prolapso objetivo, sendo, portanto, uma disfunção somática e não psicogênica. Um estudo recente mostrou associações entre as áreas hiatal, hiato genital e corpo perineal, sugerindo que a frouxidão vaginal é uma manifestação da hiperdistensibilidade do levantador do ânus. Mulheres com flacidez vaginal podem ser representativas de um estágio inicial no desenvolvimento de prolapso de órgão pélvico; no entanto, isso não foi avaliado anteriormente. Uma definição padronizada e meios para consultar pacientes em relação a tais sintomas ainda não existe. Procedimentos cirúrgicos para frouxidão vaginal com reparo posterior/perineoplastia são mais comumente recomendados, todavia, há riscos de dispareunia. Opções não cirúrgicas e com custo mais baixo podem contribuir para o tratamento da frouxidão vaginal. Entre elas destacam-se o treinamento dos músculos do assoalho pélvico e a radiofrequência. Até o momento, nenhum ensaio clínico foi desenvolvido para avaliar o papel do treinamento dos músculos do assoalho pélvico e da radiofrequência na frouxidão vaginal.

**Objetivos:** Comparar o efeito da radiofrequência microablativa fracionada isolada e do treinamento dos músculos do assoalho pélvico isolado em mulheres com queixa de frouxidão vaginal.

**Metodologia:** Trata-se de um estudo clínico, randomizado, prospectivo, controlado, paralelo e não cego. A pesquisa será desenvolvida no ambulatório de ginecologia cirúrgica da Universidade Estadual de Campinas - UNICAMP/SP. Para o estudo serão incluídas mulheres na pré-menopausa com idade ≥ 18 anos, parto vaginal, com queixa de frouxidão vaginal avaliada por pergunta direta (sim/não) e pelo *Vaginal Laxity Questionnaire* e com disponibilidade para frequentar as terapias na data e locais agendados para a realização do tratamento proposto. Os critérios de exclusão são: uso de marcapasso; doenças cardíacas descompensadas; déficit cognitivo; afecções neurológicas periféricas ou centrais; presença de qualquer tipo de câncer; presença de displasia cervical; história de infecção urinária ou vaginal ativa; doenças metabólicas descompensadas; tratamento fisioterapêutico com treinamento do assoalho pélvico prévio nos últimos 12 meses; uso de estrógeno via vaginal ou terapia hormonal oral nos últimos 6 meses, pacientes já submetidas a cirurgias de correção de prolapso ou de slings; presença de prolapso de órgão pélvico estadio 2 em diante. As participantes selecionadas serão divididas em 2 grupos de protocolos de tratamento: grupo 1 – radiofrequência e grupo 2 – treinamento dos músculos do assoalho pélvico. Após o período de intervenção, os grupos serão reavaliados em 30 e 180 dias. Para o cálculo amostral utilizamos valores da função sexual avaliada por meio do questionário *Female Sexual Function Index*. Ao se considerar um poder de estudo de 80%, um alfa de 0,05 com teste bicaudal, foi verificado que o número de participantes mínimo necessário em cada grupo será somado a um percentual de 30% de perda na amostra, totalizando 68 mulheres, sendo 34 em cada grupo (radiofrequência isolada e treinamento dos músculos do assoalho pélvico isolado). Será realizada uma análise descritiva dos dados para caracterização das participantes da pesquisa, na forma de valores de frequência absoluta e percentual (relativa) para variáveis categóricas e valores de média e desvio padrão para as variáveis numéricas. Em seguida, será realizada análise estatística de comparação e correlação dos dados obtidos a partir dos seguintes testes estatísticos: *Kolmogorov-Smirnov* para avaliação da normalidade da amostra. Dependendo dos resultados obtidos no teste de normalidade, serão utilizadas para as análises comparativas entre os grupos as Análises de Variância (se os dados apresentarem distribuição normal) ou Teste de Wilcoxon e Mann-Whitney (se os dados forem não-paramétricos). Da mesma forma, para as análises correlacionais serão utilizados os testes de *Pearson* ou *Spearman*. As variáveis categóricas serão analisadas pelos testes qui-quadrado ou pelo teste exato de Fisher. As análises estatísticas serão realizadas por meio do programa estatístico SPSS (*Statistical Package for the Social Sciences*), adotando nível de significância de 5% (p<0,05). Os dados também serão avaliados através do método de análise da variância para medidas repetidas (ANOVA for repeated measures) com o objetivo de verificar simultaneamente a influência dos 2 grupos de estudo (avaliação intergrupos) e das 2 avaliações (avaliação intragrupos) para cada uma das variáveis, afim de obter a estimação do efeito interação grupo x tempo. Caso as variáveis numéricas não apresentarem distribuição normal, serão transformadas em *ranks* ou postos*.*

**Palavras-Chave:** radiofrequência, frouxidão vaginal, fisioterapia, treinamento dos músculos do assoalho pélvico, disfunção sexual; estudo randomizado controlado

**SUMÁRIO**

1. INTRODUÇÃO8
2. JUSTIFICATIVA14
3. OBJETIVOS15

3.1 Objetivo Geral15

3.2 Objetivos Específicos15

1. HIPÓTESES16
2. MATERIAIS E MÉTODOS17

5.1 Desenho do estudo17

5.2 Tamanho Amostral17

- 1. Local da pesquisa e seleção dos sujeitos17
     1. Critérios de Inclusão18
     2. Critérios de Exclusão18
  2. Variáveis e Conceitos19
     1. Variáveis independentes19
     2. Variáveis dependentes19
     3. Variáveis descritivas21
  3. Tratamentos, técnicas, testes e/ou exames22
     1. Protocolos de tratamento22
     2. Questionários para avaliação24
     3. Avaliação funcional e morfométrica do assoalho pélvico26
  4. Coleta dos dados..................................................................................................27

5.7 Acompanhamento dos sujeitos.............................................................................28

5.8 Critérios para descontinuação, suspensão e/ou encerramento da pesquisa29

5.9 Controle de qualidade...........................................................................................29

5.10 Processamento e análise dos dados30

5.11 Divulgação dos resultados ................................................................................30

1. ASPECTOS ÉTICOS31
2. ORÇAMENTO32
3. PLANO DE ATIVIDADES E CRONOGRAMA33
4. REFERÊNCIAS34

10. **DIVULGAÇÃO DA PESQUISA ...........................................................................38**

ANEXOS39

**1-INTRODUÇÃO**

**Definição e fatores de risco**

A frouxidão vaginal é definida pela Associação Internacional de Uroginecologia (IUGA) e pela Sociedade Internacional de Continência (ICS) como uma queixa de excesso de flacidez vaginal^1^. Não existe consenso a respeito de uma definição padrão para esta situação clínica e tem ocorrido um aumento na procura para tratamento da frouxidão vaginal, principalmente na área relacionada a estética genital ^2, 3^.

Esta condição é raramente discutida entre as pacientes e seus médicos possivelmente devido à falta de tratamentos baseados em evidências, constrangimento e desconhecimento no reconhecimento desta condição ^4^. Na opinião de uroginecologistas, a frouxidão vaginal apresenta-se ainda como uma condição subnotificada com relatos de incômodos que podem afetar a função sexual e os relacionamentos ^5, 6^.

A prevalência de frouxidão vaginal é de 24% e parece estar associada à idade jovem, partos vaginais, sintomas de prolapso e prolapso objetivo, sendo portanto, uma disfunção somática e não psicogênica ^7^. Outros fatores de risco são macrossomia fetal, história de parto instrumental (fórcipe), multiparidade e alterações de tecidos conectivos. O modo como as mulheres percebem suas genitálias tem um forte e positivo impacto em sua função sexual ^8^.

Especula-se que a gravidez e o parto desempenham um papel na frouxidão vaginal ^5^. Apesar de não haver uma ligação comprovada entre frouxidão vaginal e o parto, pesquisas apontam que o parto vaginal pode resultar em lesão do assoalho pélvico ^7, 9^. O trauma do assoalho pélvico e da vagina durante a gravidez e o parto vaginal podem acarretar no alongamento do introito vaginal levando a mudanças permanentes na sensibilidade sexual e física durante o intercurso. Essas alterações promovem importante redução da qualidade de vida da mulher e da parceria^10, 11^.

Potenciais consequências associadas ao parto vaginal e que se estendem além do período pós-parto são: incontinência urinária, prolapso de órgão pélvico, dor pélvica crônica e disfunção sexual ^12-15^. Nem todas as mulheres se adaptam às mudanças psicológicas e físicas do pós-parto podendo levar a alterações na relação afetiva com a parceria^16^. Dois terços das mulheres experimentaram piora significativa da função sexual seis meses após o parto vaginal^17^. Klein *et al.* relataram que as mulheres sem trauma perineal apresentaram uma maior chance de retornarem à atividade sexual em seis semanas pós-parto em comparação com as mulheres com trauma perineal ^18^. Além disso, a dispareunia é relatada por 41 a 67% das mulheres entre dois e três meses após o parto ^12, 19-21^.

Tanto o parto vaginal como o trauma do músculo levantador do ânus estão associados ao aumento do diâmetro do hiato genital^22^. A avulsão do músculo levantador do ânus, principalmente se comprovada bilateralmente, teria algum efeito sobre a função sexual feminina ^23^. Durante o parto vaginal, o músculo puborretal é exposto a um alto grau de alongamento, com uma razão de estiramento estimada de 1,5 a 3,5 ^24, 25^. O grau de alongamento muscular parece variar de 25 a 250%^25^. Estudos de fisiologia muscular mostraram que lesão substancial, macro e microscópica, pode ocorrer se a fibra muscular esquelética for esticada para mais de 1,5 vezes a sua extensão original^26^. Não se surpreende, portanto, que 10-35% das mulheres apresentem lesão traumática do músculo puborretal na sua inserção óssea^27-29^. Isso resulta em um aumento do hiato de 20-30%,^30^ e um músculo do assoalho pélvico mais distensível e menos contrátil^22^. Em um estudo sobre a mudança periparto em dimensões hiatais, mais de 28% das primíparas foram diagnosticadas com hiperdistensão hiatal irreversível ou "microtraumas do levantador" aos 4 meses pós-parto, independente de avulsão,^31^ e sem evidência de cura após dois anos de acompanhamento^32^. O hiato genital é limitado pelo o músculo puborretal, componente do músculo levantador do ânus, e apresenta importante função na definição da zona de alta pressão vaginal ^33^.

Em um estudo que avaliou mais de 300 mulheres com frouxidão vaginal, foram encontradas associações entre a área hiatal, o hiato genital e o corpo perineal durante a manobra de Valsalva, sugerindo que a frouxidão vaginal é uma manifestação da hiperdistensibilidade do levantador do ânus e não da vagina^7^. As medidas do hiato do levantador do ânus estão fortemente associadas ao hiato genital e ao corpo perineal medidos pelo instrumento POP-Q e, portanto, não é de surpreender que este último parâmetro também estivesse fortemente associado ao sintoma da frouxidão vaginal^7^. Mulheres com flacidez vaginal podem ser representativas de um estágio inicial no desenvolvimento de prolapso de órgão pélvico; no entanto, isso não foi avaliado anteriormente^5^. Uma definição padronizada e meios para consultar pacientes em relação a tais sintomas ainda não existe^5^.

Os níveis séricos de estradiol na mulher em idade reprodutiva variam de 30 a 300 pg / mL, dependendo da fase do ciclo menstrual. Mulheres na pós-menopausa têm esse nível reduzido em mais de 90% para uma média de 6,5 pg / mL ^34^. Mudanças profundas ocorrem na mucosa vulvovaginal e urogenital com a perda da estimulação estrogênica ^35^. O hipoestrogenismo também resulta em alterações do tecido conectivo, mudanças na estrutura pélvica e declínio da qualidade do colágeno^36^. A idade e alterações hormonais geram uma deterioração e relaxamento do tecido conectivo e das fibras colágenas, diminuindo o suporte dos órgãos pélvicos devido ao decréscimo do diâmetro e do número de fibras musculares estriadas periuretrais e do assoalho pélvico^37^. Essa fisiopatologia é importante para o entendimento de alguns tipos de tratamento como a radiofrequência.

**Diagnóstico e tratamento**

A redução da sensação vaginal durante a relação sexual pode estar relacionada a danos anatômicos no corpo perineal, prolapso no estágio 1, frouxidão do canal vaginal ou introito, dano subjacente aos nervos e tecido conjuntivo durante a gravidez e o parto ou, potencialmente, uma combinação desses fatores^38^.

O diagnóstico da frouxidão vaginal é baseado no auto relato das pacientes. Uma história médica abrangente, um exame físico e uma avaliação psicossexual são os passos iniciais para identificação apropriada de pacientes com frouxidão vaginal^39^.

Um instrumento que vem sendo usado em pesquisas clínicas para auxiliar na identificação e no grau da frouxidão é o “ *Vaginal Laxity Questionnaire*”. Este instrumento de avaliação autorreferida de frouxidão vaginal usa uma escala de sete pontos associada à uma pergunta: Como você avaliaria seu nível atual de frouxidão vaginal? ou frouxidão durante a relação sexual? ^40^.

A anatomia desempenha um papel importante na compreensão das diferentes estruturas envolvidas no suporte pélvico^41^. A cintura pélvica é composta por várias camadas de músculos e fáscias de suporte que se interligam e se sobrepõem, contribuindo para o suporte global e o funcionamento normal da vagina e de suas estruturas adjacentes^42, 43^. A história e o exame físico determinarão se a paciente é um candidata a procedimentos vaginais ou a uma abordagem de reconstrução vaginal mais complexa^44^. Antes que se possa manejar adequadamente essas pacientes, é importante entender a complexa mecânica estrutural da falência posterior da parede vaginal^45^. A falha da parede posterior pode envolver falha do suporte do corpo perineal e dos músculos levantadores do ânus, o que pode resultar em um hiato genital alargado^45^. Os músculos levantadores fornecem uma ação tônica e cefálica que mantém o hiato genital fechado a uma dimensão normal em resposta à pressão. Se os levantadores estiverem enfraquecidos ou lesionados, ou se os anexos fasciais da parede vaginal posterior estiverem acometidos (retocele), ocorre uma descida descendente do corpo perineal e o hiato se abre^45^. O enfraquecimento da fáscia endopélvica em compartimento anterior poderia ser mais estudado para associar a hipermobilidade uretral e consequente incontinência urinária de esforço com a frouxidão vaginal^46^.

O diagnóstico de prolapso de órgão pélvico exige evidências clínicas claras, começando com uma mulher com sintomas relacionados ao “deslocamento para baixo” de um órgão pélvico. Os sintomas são geralmente piores em situações em que a gravidade pode piorar o prolapso (por exemplo, após longos períodos em pé ou exercício) e melhores quando a gravidade não é um fator, por exemplo, deitada em posição supina. Novamente, os sintomas podem ser mais perceptíveis em momentos de esforço abdominal, por exemplo, defecação^47^.

A função e a contratilidade dos músculos do assoalho pélvico são avaliadas por meio da Escala de Oxford Modificada por Laycock (1994)^48^. Essa escala é classificada de zero a 5, sendo zero-sem função perineal objetiva e 5- contração de intensidade ótima e elevação cranial da parede vaginal^48^.

Procedimentos cirúrgicos para frouxidão vaginal com reparo posterior/perineoplastia são mais comumente recomendados, todavia, 83% dos uroginecologistas entrevistados reportaram preocupação potencialmente importante com casos de dispareunia^5^. Nos últimos anos houve um número crescente de vários tipos de cirurgias vulvovaginais comercializadas como formas de melhorar aparência ou gratificação sexual. Entre eles destacam-se o chamado rejuvenescimento vaginal, vaginoplastia designer, revirginização e amplificação do ponto G. Alguns procedimentos, como o rejuvenescimento vaginal, parecem ser modificações dos procedimentos cirúrgicos vaginais tradicionais. Outros procedimentos são realizados para alterar o tamanho ou a forma do lábio maior ou lábio menor. Revirginização envolve reparo himenal em uma tentativa de aproximar o estado virginal. Amplificação do ponto G envolve a injeção de colágeno na parede anterior da vagina. Apesar de serem realizados, a segurança e a eficácia destes procedimentos a longo prazo ainda não foram documentados ^49^.

Uma opção não cirúrgica para o tratamento da frouxidão vaginal inclui o treinamento da musculatura do assoalho pélvico que foi inicialmente recomendado para o tratamento de incontinência urinária ^4^. A função do músculo do assoalho pélvico parece ter um papel importante na função sexual feminina, e a contração do músculo elevador do ânus parece aumentar a resposta sexual ^50^.

A contração dos músculos do assoalho pélvico também desempenha um papel importante na resposta orgástica feminina. Mulheres com músculos fracos que recebem reabilitação do assoalho pélvico e fortalecem os músculos dessa região percebem um efeito positivo em sua vida sexual ^51^. O treinamento dos músculos do assoalho pélvico (TMAP) demonstrou ser um tratamento eficaz para a dispareunia^52^. Geralmente, é recomendado como tratamento de primeira linha, já que tem sido associado a mínimos efeitos adversos e baixo custo ^53^.

Outra possibilidade terapêutica não cirúrgica para tratar a frouxidão vaginal é a radiofrequência (RF). Um estudo piloto para o uso da radiofrequência para o tratamento da frouxidão vaginal mostrou que o tratamento foi bem tolerado pelas participantes e apresentou melhora subjetiva do estreitamento vaginal, função sexual e diminuição do desconforto sexual ^4^.

  A radiofrequência é gerada pelo campo elétrico resultante da oscilação da corrente elétrica que, por sua vez, induz o movimento translacional de átomos e moléculas carregadas e dificulta a rotação de moléculas polares^54^. Esta molécula em movimento, é em grande parte responsável pela capacidade de calor e aumento da temperatura local.  Na presença de um campo elétrico, as moléculas orientam-se ao longo da direção do campo, mas devido à viscosidade da água, é necessária energia para girar os dipolos resultando em transferência de energia para o tecido. A resistência ou impedância converte corrente elétrica para energia térmica gerando calor em relação à quantidade de tempo atual e exposição. Consequentemente, a energia é dispersa em volumes tridimensionais de tecido em profundidades controladas ^54^.

A energia da radiofrequência tem uma longa história de uso no tecido mucoso da vagina e da pele ^55-57^. Através da criação de calor via impedância à medida que a corrente elétrica é conduzida através do tecido vaginal, a estimulação de fibroblastos ocorre e o resultado terapêutico é alcançado ^58^. O intervalo de temperatura do tecido alvo situa-se entre 40º e 47 ℃. A efetividade da radiofrequência na umidade natural foi demonstrada no estudo histológico de radiofrequência no tecido vaginal de ovelhas^59^. A radiofrequência também foi eficaz para o rejuvenescimento vulvovaginal ^4^. Um estudo utilizando a radiofrequência de baixa energia para frouxidão vaginal introital em mulheres na pré-menopausa apontou melhorias tanto na frouxidão quanto na função sexual. Os efeitos foram mantidos por 12 meses e nenhum evento adverso foi relatado ^10^.

Até o momento, nenhum ensaio clínico foi desenvolvido para avaliar o papel do treinamento dos músculos do assoalho pélvico e da radiofrequência na frouxidão vaginal.

**2. JUSTIFICATIVA**

Os procedimentos cirúrgicos para o reparo da frouxidão vaginal são comumente recomendados mas podem acarretar em dispareunia, importante fator adverso na função sexual feminina^5^. Outros procedimentos cirúrgicos são apontados, como é o caso da cirurgia de revirginização com reparo himenal, no entanto, a segurança e eficácia desse tratamento ainda não foi documentada.

Opções não cirúrgicas que ofereçam mínimos efeitos adversos podem ser indicadas para o tratamento da frouxidão vaginal com custos menores que os procedimentos cirúrgicos. O treinamento dos músculos do assoalho pélvico apresenta-se como primeira linha no tratamento na incontinência urinária. Já a radiofrequência apresentou melhorias tanto na frouxidão vaginal quanto na função sexual mantidos por 12 meses em poucos trabalhos já discutidos anteriormente. Comparativamente a outros tratamentos conservadores como o laser, a RF apresenta custos mais econômicos, e possibilidade de atingir populações com menor poder aquisitivo para conseguir tratar.

Assim, ao avaliarmos o comportamento tanto da radiofrequência como do treinamento dos músculos do assoalho pélvico na frouxidão vaginal, poderemos contribuir para a indicação dessas modalidades para melhoria da qualidade de vida sexual feminina.

**3. OBJETIVOS**

- 1. **Objetivo Geral**

Comparar o efeito da RF microablativa fracionada isolada e do TMAP isolado em mulheres com queixa de frouxidão vaginal.

**3.2 Objetivos Específicos**

- Comparar o efeito da RF isolada e do TMAP isolado sobre a função sexual.
- Comparar o efeito da RF isolada e do TMAP isolado sobre os sintomas urinários.
- Comparar o efeito da RF isolada e do TMAP isolado sobre a contratilidade e função dos músculos do assoalho pélvico.
- Comparar o efeito da RF isolada e do TMAP isolado sobre a escala de frouxidão vaginal.
- Comparar o efeito da RF isolada e do TMAP isolado sobre os níveis de ansiedade e depressão em mulheres com frouxidão vaginal.

**4. HIPÓTESES**

- As mulheres submetidas ao tratamento com RF deverão ter melhora subjetiva semelhante ao das mulheres tratadas com o TMAP para a frouxidão vaginal.
- A qualidade de vida das mulheres com os dois tratamentos será semelhante.
- O tratamento com RF apresentará presença de efeitos adversos em comparação com o TMAP, está última é geralmente isenta de efeitos colaterais.
- O tratamento com RF apresentará melhora da função sexual em mulheres com frouxidão vaginal de forma similar com o TMAP.

**5. MATERIAIS E MÉTODOS**

**5.1 Desenho do estudo**

Estudo clínico, randomizado, prospectivo e controlado, paralelo,não-inferioridade, não cego.

**5.2 Tamanho amostral**

O cálculo amostral foi baseado utilizando como base o estudo de Krychman *et al*. ^40^, que demonstrou que a terapia com RF foi associada à importante melhora clínica e estatisticamente significativa da função sexual em mulheres com frouxidão vaginal, quando realizada análise dos dados em um grupo contendo 73 pacientes. Para o cálculo da amostra do presente estudo, utilizamos valores da função sexual avaliada por meio do questionário FSFI. Houve um aumento de 7 pontos no score do FSFI no grupo tratado com radiofrequência e um aumento de 3 pontos no grupo controle. Ao se considerar um poder de estudo de 80%, um alfa de 0,05 com teste bicaudal, foi verificado que o número de participantes mínimo necessário em cada grupo será somado a um percentual de 30% de perda na amostra, totalizando 68 mulheres, sendo 34 em cada grupo (RF isolada e TMAP isolado).

A randomização será realizada através de programa de computador, na proporção 1:1, em dois blocos. Os números correspondentes aos grupos de estudo (1. Grupo Radiofrequência e 2. Grupo Treinamento dos Músculos do Assoalho Pélvico) serão colocados dentro de envelopes opacos que serão abertos pela mulher após assinarem o termo de consentimento e serem submetidas a avaliação pré-intervenção.

**5.3 Local da pesquisa e seleção dos sujeitos**

A pesquisa será desenvolvida no ambulatório de uroginecologia da Faculdade de Ciências Médicas e no Ambulatório de Fisioterapia do Centro de Atenção à Saúde Integral da Mulher – CAISM da Universidade Estadual de Campinas – UNICAMP.

A seleção das voluntárias será realizada por meio do encaminhamento de mulheres com diagnóstico clínico de frouxidão vaginal provenientes do Ambulatório de Uroginecologia e do Centro de Atenção à Saúde Integral da Mulher – CAISM / UNICAMP e também através da divulgação da pesquisa por meio das mídias sociais e imprensa para demanda espontânea das voluntárias de fevereiro de 2020 a julho de 2021.

Através de palestras, todas as mulheres serão informadas sobre os procedimentos de análise, os critérios e tratamento a que serão submetidas, inclusive a possibilidade de não participar do estudo. As que aceitarem participar deverão assinar um Termo de Consentimento Livre e Esclarecido, aprovado pelo Comitê de Ética em Pesquisa (Anexo 10).

5.3.1 Critérios de inclusão:

Serão incluídas mulheres com idade ≥ 18 anos, parto vaginal, com queixa de frouxidão vaginal avaliada por pergunta direta (sim/não) e pelo *Vaginal Laxity Questionnaire* (VLQ) (Anexo 1), e com disponibilidade em frequentar as terapias na data e locais agendados para a realização do tratamento proposto.

5.3.2. Critérios de exclusão:

Serão excluídas, de ambos os grupos, aquelas com:

- Uso de marcapasso;

- Doenças cardíacas descompensadas;

- Déficit cognitivo;

- Afecções neurológicas periféricas ou centrais;

- Presença de qualquer tipo de câncer;

- Presença de displasia cervical;

- História de infecção urinária ou vaginal ativa

- Doenças metabólicas descompensadas;

- Pacientes em tratamento fisioterapêutico com treinamento do assoalho pélvico;

- Pacientes em uso de estrógeno via vaginal/oral nos últimos 6 meses;

- Pacientes já submetidas a cirurgias de correção de prolapso ou de slings.

- Pacientes com prolapso de órgão pélvico estadio 2 em diante;

- Grau de força de contração dos músculos do assoalho pélvico igual a zero de acordo com a Escala Modificada de Oxford.

**5.4 Variáveis e conceitos**

5.4.1 Variáveis independentes (grupos de intervenção):

- Aplicação de Radiofrequência: aplicação de radiofrequência microablativa via vaginal com conversão para energia térmica que visa a degradação de fibras de colágeno e novo modelamento destas promovendo fortalecimento das estruturas de sustentação do assoalho pélvico.

- Treinamento dos Músculos do Assoalho Pélvico (TMAP): tratamento fisioterápico que visa ao fortalecimento das estruturas de sustentação do assoalho pélvico

- Avaliação da Resposta Global: essa escala subjetiva será adaptada para a presente pesquisa de acordo com o estudo de Millheiser *et al*. ^4^ Trata-se de uma escala de sete pontos com resposta a seguinte pergunta: Como você está se sentindo agora ( níveis de frouxidão/aperto vaginal e satisfação sexual) comparado com o início do tratamento? 1-melhorou acentuadamente, 2-melhorou moderadamente, 3-melhorou ligeiramente, 4-nenhuma mudança, 5-um pouco pior, 6-moderadamente pior, 7-muito pior.

- - 1. Variáveis dependentes:

- *Frouxidão vaginal*: condição clínica referente à sensação de “frouxidão” da abertura vaginal percebida pela mulher e/ou parceria sexual durante a penetração^5^. A frouxidão vaginal será avaliada clinicamente, por meio de pergunta direta (sim/não) e pelo *Vaginal Laxity Questionnaire* (VLQ) (Anexo 1).

*- Função sexual feminina:*  condições clínicas das mulheres ligadas ao ato sexual investigadas por meio do questionário validado *Female Sexual Function Index* (FSFI) ^60^ (Anexo 2). A dispareunia será avaliada por meio da escala *Marinoff Scale* ^61^ (Anexo 3).

*- Sintomas vaginais:* condições clínicas relacionadas a vagina investigada por meio do questionário validado pela *International Consultation on Incontinence Questionnaire - Vaginal Symptoms* (ICIQ-VS)^62^ (Anexo 4).

- *Angústia sexual e depressão*: o sofrimento sexual será medido pela escala *Female Sexual Distress Scale-Revised* ^63^ (Anexo 5).

*- Incontinência urinária (IU):* perda urinária durante situações de esforço (tais como tosse, espirro, salto); investigada por meio do questionário *International Consultation on Incontinence Questionnaire Short Form* (ICIQ UI-SF), o qual permite qualificar e quantificar a perda urinária além de investigar seu impacto na qualidade de vida^64^ (Anexo 6).

- *Mobilidade uretral:* diferença do posicionamento da uretra em repouso e durante a manobra de Valsalva Máxima durante a avaliação clínica.

*- Prolapso de órgão pélvico (POP*): descenso da parede vaginal anterior e/ou posterior, assim como do ápice da vagina (útero ou cúpula vaginal após histerectomia), investigado por meio do instrumento *Pelvic Organ Prolapse Quantification* (POP-Q) para avaliação e estadiamento do prolapso^47^ (Anexo 7 ).

- *Força e função dos músculos do assoalho pélvico*: intensidade de pressão que a musculatura perineal exerce sobre os dois dedos do examinador durante o toque vaginal. Paciente posicionada em decúbito dorsal e com os pés apoiados na maca será orientada a realizar uma contração voluntária máxima da musculatura, graduada pela Escala Modificada de Oxford, em 5 níveis: zero ou ausente =  não se observa sinais de contração muscular; 1 = força muscular mínima (sinais de discreta contratilidade, sem movimentos da articulação); 2 = força muscular fraca (mobilidade em todos os sentidos normais, com eliminação da gravidade); 3 = força muscular regular (movimentos de amplitude normal contra a ação da gravidade); 4 = boa força muscular (mobilidade integral contra a ação da gravidade e de certo grau de resistência); 5 = força muscular normal (mobilidade completa contra resistência acentuada e contra a ação da gravidade)^48^ (Anexo 8). A morfometria dos músculos do assoalho pélvico será realizada utilizando o aparelho de ultrassonografia transperineal. As medidas serão realizadas em repouso, manobra de Valsalva e contração muscular do assoalho pélvico com a paciente em posição litotômica. As indicações desse método de avaliação estão de acordo com Dietz *et.al*^65^ (Anexo 8).

- Espessura Vaginal: A espessura vaginal será avaliada em seus terços proximal, médio e distal usando duas abordagens – transabdominal e transvaginal^66, 67^. Os transdutores 4C-D 2 - 5 MHz transabdominal e 5 - 9 MHz transvaginal serão usados (Anexo 8).

- - 1. Variáveis descritivas:

- *Idade*: expressa em número absoluto, em anos, informada pela paciente;

*- Cor da pele:* cor da pele declarada pela própria mulher e categorizada em branca e não branca;

*-Grau de escolaridade:* grau de estudo da paciente especificada em ensino fundamental completo ou incompleto, ensino médio completo ou incompleto e ensino superior completo ou incompleto;

*- Frequência de relação sexual com penetração:* frequência semanal de ato sexual com penetração vaginal apresentada em números absolutos;

*- Tabagismo:* paciente com vício de qualquer tipo de tabaco, autodeclarada tabagista, ex-tabagista ou não tabagista;

- *Paridade*: número de gestações relatado pela mulher e expresso em número absoluto, diferenciando parto via vaginal, parto cesárea e aborto.

- *Menopausa:* idade, em anos, da ocorrência da última menstruação.

- *Índice de massa corpórea (IMC):* medida do índice de massa corporal, através do cálculo da razão entre o peso e o quadrado da altura da paciente, classificado em baixo (<20); normal (20-25); sobrepeso (26-30); obesidade moderada (31-35); obesidade severa (36-40); obesidade mórbida (41-50).

- *Sedentarismo:* definido como a ausência de atividade física por pelo menos 10 minutos contínuos durante a semana, categorizado em presente ou ausente.

- *Comorbidades:* avaliação de antecedentes mórbidos, obtidos através de entrevista e classificados em: cardiovascular, respiratório, neurológica, endócrino, ortopédica, urológica, ginecológica, psiquiátrica e reumatológica.

**5.5 Tratamentos, técnicas, testes e exames**

5.5.1 Protocolos de tratamento

Para este estudo as pacientes serão divididas em 2 grupos de protocolos de tratamento:

*a) Grupo Radiofrequência*

As participantes serão submetidas a três aplicações, mensais totalizando 12 semanas de tratamento. Será utilizado o aparelho Wavetronic 6000 Touch com o sistema Megapulse HF FRAXX (Loktal Medical Electronics, São Paulo, Brasil), equipado com circuito eletrônico de fracionamento de energia, conectado a uma caneta vaginal com 64 microagulhas de 200µ de diâmetro e 1mm de comprimento, montadas em um suporte de teflon e divididas em uma matriz de oito colunas, com oito agulhas cada. Ao pressionar o pedal de disparo, essas 64 agulhas não são energizadas simultaneamente e a liberação de energia é randomizada em colunas de oito agulhas em uma sequência predefinida, que não permite que duas colunas adjacentes disparem em sequência, prevenindo a soma térmica das colunas (controle de disparo fracionado exclusivo Smart Shoot). Isso permite o resfriamento entre os pontos e a preservação de tecidos adjacentes aos pontos vaporizados, para que ocorram a neocolagênese e a neoelastogênese, por meio de estimulação fibroblástica. Cada disparo da caneta realiza 64 microablações na mucosa ^68^.

A participante será posicionada em decúbito dorsal com membros inferiores flexionados e abduzidos, em posição de litotomia.

No vestíbulo e na abertura vaginal, será aplicada lidocaína spray 10% 3 minutos antes do procedimento. Será então introduzido um espéculo vaginal descartável, e posteriormente será realizada a antissepsia com clorexidina aquosa 0,2%, a limpeza com solução salina estéril 0,9% para remover o conteúdo vaginal excedente com gaze. A aplicação sequencial de RF microabativa nas paredes vaginais sob visão direta, será realizada movendo-se o espéculo quando necessário. No vestíbulo, a aplicação ocorrerá apenas no introito vaginal, sem incluir clitóris, prepúcio do clitóris e lábios menores. O eletrodo será sempre mantido paralelo, encostando levemente na mucosa a cada disparo. O tempo médio de procedimento será de 15 a 20 minutos ^68^.

Para os cuidados pós-tratamento, será recomendado o uso de solução de dexpantenol 5% na abertura vaginal, duas a três vezes por dia, durante 2 a 5 dias, e interrupção de relações sexuais por 10 dias^68^.

Todas as aplicações serão realizadas pela pesquisadora colaboradora.

*b) Grupo TMAP*

O protocolo de treinamento muscular do assoalho pélvico será realizado pela pesquisadora colaboradora no Centro de Atenção Integral a Saúde da Mulher – Caism – Hospital da Mulher Prof. Dr. José Aristodemo Pinotti. Serão realizadas, em grupos de 6 a 10 pacientes, 1 sessão semanal por 12 semanas, totalizando 12 sessões, com duração de 60 minutos cada, de acordo com o protocolo publicado por Dumoulin *et al.*^69^ e Bo *et al*.^70^. Este protocolo consiste em exercícios de mobilidade pélvica, fortalecimento, resistência e coordenação e serão realizados em todas as sessões em diferentes, juntamente com as contrações do assoalho pélvico, que consistem em contrações moderadas (3 repetições/6 segundos; 3 repetições/8 segundos; 3 repetições/10 segundos), contrações máximas (6 repetições/ 6 segundos; 8 repetições/8 segundos; 10 repetições/10 segundos e contrações rápidas (2 repetições/8 contrações, 2 repetições/10 contrações). Os exercícios serão incrementados em dificuldade, repetição e duração ao longo das 12 semanas. Será oferecido para as participantes um diário de exercícios para que estimule o treinamento muscular domiciliar, seguindo o mesmo esquema de contrações dos músculos do assoalho pélvico.

        Para este estudo serão utilizados os seguintes equipamentos:

- Wavetronic 6000 Touch com o sistema Megapulse HF FRAXX (Loktal Medical Electronics, São Paulo, Brasil), equipado com circuito eletrônico de fracionamento de energia, conectado a uma caneta vaginal com 64 microagulhas de 200µ de diâmetro e 1mm de comprimento, montadas em um suporte de teflon e divididas em uma matriz de oito colunas, com oito agulhas cada.

- Equipamento de ultrassonografia transperineal GE Voluson 730 Expert® (GE Medical System Kretz-technik GmbH and Co OHG, Zipf, Austria) com sonda 3D / 4D de 2 a 6 MHz convexa RAB4-8L, com a função de registrar a morfometria dos músculos do assoalho pélvico. Os transdutores 4C-D 2 - 5 MHz transabdominal e 5 - 9 MHz transvaginal serão usados para a espessura vaginal.

5.5.2. Questionários para avaliação

Vaginal Laxity Questionnaire (VLQ): um questionário contendo uma pergunta (como você considera o nível da sua frouxidão ou fortalecimento/apertamento vaginal?) sobre a frouxidão vaginal com sete respostas ordenadas (muito frouxa=0, moderadamente frouxa=1, levemente frouxa=2, nem frouxa nem apertada=3, levemente apertada=4, moderamente apertada=5, muito apertada=6 ^40^.

Marinoff Scale: Escala de Dispareunia de Profundidade (EDP) que avalia a classificação de dor durante a penetração de profundidade do pênis na relação sexual. O instrumento elaborado possui graduação, sendo zero (ausência de dor durante a relação sexual); um (dor leve, que não obriga a interromper a relação sexual); dois (dor moderada, que dificulta, mas não obriga a interromper a relação sexual) e três (dor intensa, que obriga a interromper a relação sexual)^61^.

FSFI (Female Sexual Function Index): instrumento breve e multidimensional para avaliar a função sexual em mulheres. O questionário foi desenvolvido e validado por Rosen *et al.* e consiste em 19 itens que investiga a resposta sexual ao longo das últimas quatro semanas e o rendimento em seis domínios: desejo sexual, excitação, lubrificação, orgasmo, satisfação e dor ^60^. A validação em português ocorreu em 2008 por Thiel *et al.* ^71^. As respostas são pontuadas de acordo com a soma dos itens que compõem cada domínio (escore simples) e multiplicadas pelo fator do domínio gerando o escore ponderado ^71^. A pontuação máxima é 36 pontos, somando-se o total de cada domínio.

Wiegel *et al.* propôs uma pontuação de corte para diferenciar as mulheres com ou sem disfunção sexual no valor de 26,55 ^72^. As mulheres que apresentaram pontuações abaixo do valor de corte foram classificadas como sexualmente disfuncionais.

analisa a resposta sexual quanto a desejo, excitação, lubrificação vaginal, orgasmo, satisfação sexual e dor. O escore total é a soma dos escores para cada domínio multiplicada pelo fator correspondente e pode variar de ‘2’ a ‘36’, considerando risco para disfunção sexual um escore total menor ou igual a ‘26’.

FSDS-R – Female Sexual Distress Scale-Revised: a escala mede o sofrimento sexual com um escore composto maior ou igual a 11 traduzindo para um diagnóstico de sofrimento sexual. Pontuações menores que 11 indicam que não há angústia ^63^. A angústia sexual é caracterizada por um conjunto de sentimentos (por exemplo, infelicidade, culpa, frustração, estresse, preocupação) e emoções que os indivíduos têm sobre sua sexualidade. Difere da disfunção sexual relacionada a sintomas da função sexual, como excitação, orgasmo e dor, separados das emoções^63^.

ICIQ-SF (International Consultation on Incontinence Questionnaire Short-Form): validado na língua portuguesa por Tamanini *et al.* , sendo considerado um questionário simples, breve e auto-administrável, capaz de avaliar de forma rápida e eficaz o impacto da incontinência urinária na qualidade de vida dos pacientes, além de qualificar a perda urinária em ambos os sexos. É composto por quatro questões que avaliam a freqüência, a gravidade e o impacto da incontinência urinária, além de um conjunto de oito itens de auto-diagnóstico, relacionados às causas ou situações de incontinência urinária vivenciadas pelos pacientes. Sua pontuação pode variar de 0 a 21, sendo maior o comprometimento, quanto maior for o valor total ^64^.

ICIQ-VS (International Consultation on Incontinence Questionnaire - Vaginal Symptoms) validado para a língua portuguesa por Tamanini *et al*. é um questionário breve que avalia a presença e a intensidade os sintomas vaginais, bem como sua relação com a qualidade de vida ^62^.

POP-Q - Pelvic Organ Prolapse Quantification: Sistema frequentemente usado por uroginecologistas e a “*International Continence Society*” (ICS) recomenda a descrição e o estadiamento do POP por meio desse instrumento ^73, 74^.

A classificação do estadiamento é definida como ^47^:

Estadio 0: Não há prolapso demonstrado.

Estadio I: A maior parte distal do prolapso está a mais de 1 cm acima do nível do hímen.

Estadio II: A porção mais distal do prolapso situa-se entre 1 cm acima do hímen e 1 cm abaixo do hímen.

Estadio III: A porção mais distal do prolapso está mais de 1 cm para além do plano do hímen, mas evertido pelo menos 2 cm a menos que o comprimento vaginal total.

Estadio IV: Eversão completa ou eversão de até 2 cm do comprimento total do trato genital inferior.

O hímen é o ponto de referência usado para a descrição do prolapso quantitativo e representa o ponto zero. As pacientes serão examinadas em decúbito dorsal e as medidas realizadas em centímetros com o auxílio de uma régua graduada descartável.

Seis pontos anatômicos serão avaliados conforme orientação da ICS ^47^ (dois na parede vaginal anterior – Aa e Ba, dois na parede vaginal posterior – Ap e Bp e dois pontos na vagina superiormente – C e D). Será medido também o hiato genital (do centro do meato uretral externo até a margem posterior do hímen), o comprimento vaginal total (comprimento da vagina do fórnice posterior até o hímen quando o ponto C ou D é reduzido para sua posição normal) e o corpo perineal (da margem posterior do hímen até orifício anal). Todos os pontos serão medidos em Valsalva máxima, exceto o comprimento vaginal total ^47^.

A ICS definiu clinicamente o POP significativo em estadio II ou superior^47, 74^.

5.5.3 Avaliação funcional e morfométrica do assoalho pélvico

A paciente será posicionada em decúbito dorsal, com os membros inferiores fletidos e pés apoiados na maca. A avaliação da contratilidade dos músculos do assoalho pélvico, será realizado primeiramente através de palpação bi-digital, introduzindo de 2-3cm os dedos indicador e médio no introito vaginal, realizando a abdução dos dedos e solicitando que a paciente realize a contração voluntária máxima, apertando e realizando movimento cranial dos dedos do avaliador. A contratilidade muscular será graduada de acordo com a escala modificada de Oxford (0-5) ^48^.

A força e a resistência dos músculos do assoalho pélvico também serão registradas utilizando o perineômetro. As medidas serão registradas através de uma sonda introduzida no canal vaginal e ligada a um manômetro digital em cmH_2_O, que fornece o pico de pressão, tempo de contração e velocidade de contração^75^. O protocolo de avaliação consistirá em três contrações voluntárias máximas com intervalo de 30 segundos entre cada medida, registradas pelo probe vaginal. A média das três contrações será considerada para análise. A resistência será medida após o teste de contração voluntária máxima. As participantes serão solicitadas a manter a contração dos músculos do assoalho pélvico pelo tempo que puderem. Elas não serão interrompidas a menos que as medidas de pressão atingirem zero ou se caso relatem não conseguirem segurar a contração por mais tempo. O tempo da contração será medido em segundos e usado para análise de dados.

O protocolo de avaliação morfométrica dos músculos do assoalho pélvico consistirá de medidas em repouso, durante a contração máxima dos músculos do assoalho pélvico e em manobra de Valsalva. Antes de realizar o exame, as pacientes serão solicitadas a esvaziarem a bexiga e posicionadas em litotomia com os membros inferiores fletidos e apoiados na maca. Para o exame será usado transdutor convexo RAB4-8L, coberto com gel à base de água, protegido com protetor de látex e posicionado longitudinalmente sobre o introito vaginal. Cada manobra será realizada duas vezes e o volume ultrassonográfico com maior deslocamento angular será considerado para análise. Os parâmetros avaliados seguirão o protocolo de Cyr *et al.^76^*, com análise no plano médio-sagital e no plano axial (dimensões hiatais mínimas): a posição do colo vesical (posições do eixo-x e do eixo-y); o ângulo da placa do elevador, o ângulo anorretal, espessura do músculo levantador do ânus e a área do hiato do elevador em cm^2^ (medida anteroposterior e diâmetros transversos latero-lateral). Os dados de ultrassonografia serão analisados off-line com o software (4D View, versão 10.2; GE Healthcare) por um observador cego. A espessura vaginal será avaliada em seus terços proximal, médio e distal usando duas abordagens - transabdominal e transvaginal^66, 67^.

**5.6 Coleta dos dados**

A triagem das participantes com frouxidão será realizada no Ambulatório de Uroginecologia pela pesquisadora colaboradora. Todas as participantes serão orientadas quanto aos objetivos da proposta e os métodos de avaliação pelos quais serão submetidas. As mulheres que aceitarem participar do estudo deverão assinar um Termo de Consentimento Livre e Esclarecido - TCLE (Anexo 10), em duas vias assinadas, uma ficando sob os cuidados da pesquisadora e outra sob os cuidados da voluntária.

As mulheres que aceitarem participar do estudo serão submetidas ao processo de avaliação previamente agendado. Neste momento será realizada coleta de dados sociodemográficos, aplicados os questionários (anexos 1, 2, 3, 4, 5, 6 e 8), realizada avaliação funcional e morfométrica dos músculos do assoalho pélvico. Estes procedimentos serão realizados no ambulatório de ginecologia pelos pesquisadores participantes da pesquisa.

As 68 mulheres serão então randomizadas para um dos dois grupos de pesquisa. O processo de randomização será feito através de programa de randomização pelo estatístico a medida que as pacientes forem incluídas na pesquisa. O programa selecionará o grupo que a mulher irá pertencer (RF isolado e TMAP isolado). Cada grupo será composto por 34 mulheres. As mulheres do grupo RF isolado serão orientadas a comparecerem uma vez por mês por 3 meses (totalizando 3) no ambulatório de cirurgia ginecológica para aplicação de RF. As mulheres do grupo TMAP isolado serão convidadas a comparecerem no setor de fisioterapia uma vez por semana por 12 semanas. A aplicação da RF será feita pela pesquisadora colaboradora, após treinamento prévio com uma médica que já realiza o procedimento, em pelo menos 10 casos, para adquirir prática na realização da técnica.

As mulheres serão orientadas a retornarem 30 dias e 180 dias após última aplicação de RF ou da última sessão de TMAP. Nestes retornos, serão aplicados novos questionários e serão realizadas novas avaliações da função do assoalho pélvico e prolapso de órgão pélvico.

No total, as mulheres que participarem do grupo TMAP deverão comparecer ao serviço 15 vezes (para tratamento, avaliação e reavaliação) e as que realizarem a RF deverão comparecer 6 vezes (para tratamento, avaliação e reavaliação).

- 1. **Acompanhamento dos sujeitos**

As participantes do estudo, ao serem avaliadas, receberão orientações fisioterapêuticas pertinentes à sua condição e serão acompanhadas durante todo o processo de tratamento e após 30 e 180 dias.

**5.8 Critérios para descontinuação, suspensão e/ou encerramento da pesquisa**

A descontinuação, suspensão e/ou encerramento da pesquisa ocorrerão mediante a verificação de índices significativos de desconforto durante aplicação da RF microablativa vaginal, avaliação da musculatura do assoalho pélvico e prolapso de órgão pélvico, bem como pela ocorrência significativa de eventos como: infecção do trato urinário (ITU), vulvovaginites e irritação e lesão vaginal grave. Nestes casos, será oferecido tratamento médico adequado.

As mulheres serão descontinuadas se tiverem qualquer falta nas sessões de radiofrequência e/ou a presença nas sessões de fisioterapia não atingir 80%.

**5.9 Controle de qualidade**

Durante a etapa de coleta dos dados, serão adotados como controle de qualidade os seguintes cuidados:

- A randomização com sorteio e convocação para os tratamentos será realizada por uma única pessoa;

- Os questionários serão aplicados por um único pesquisador, pré e pós tratamento, o mesmo será cego em relação a qual tipo de terapêutica foi realizada;

- A avaliação dos músculos do assoalho pélvico será realizada por um único avaliador experiente na avaliação dos músculos do assoalho pélvico feminino, pré e pós terapia;

- A aplicação da RF a microablativa via vaginal será aplicada por um único pesquisador treinado para a realização e seguindo o mesmo protocolo.

        Durante a etapa de avaliação dos dados, serão adotados os seguintes cuidados:

- O banco de dados será duplamente digitado por dois pesquisadores independentes e, caso haja discordância entre algum dado, haverá revisão do mesmo;

- O estatístico será cego com relação aos grupos;

**5.10 Processamento e análise dos dados**

A análise dos dados será precedida pela elaboração de um banco de dados no aplicativo M*icrosoft Office Excel* 2010, utilizado para codificação das variáveis em um dicionário de dados e para validação mediante dupla entrada (digitação) dos dados.

Inicialmente, será realizada uma análise descritiva dos dados para caracterização das participantes da pesquisa, na forma de valores de frequência absoluta e percentual (relativa) para variáveis categóricas e valores de média e desvio padrão para as variáveis numéricas.

Em seguida, será realizada análise estatística de comparação e correlação dos dados obtidos a partir dos seguintes testes estatísticos: *Kolmogorov-Smirnov* para avaliação da normalidade da amostra, e, dependendo dos resultados obtidos no teste de normalidade serão utilizados para as análises comparativas entre os grupos a Análises de Variância se os dados apresentarem distribuição normal ou Teste de *Wilcoxon* e *Mann-Whitney* se os dados forem não-paramétricos Da mesma forma, para as análises correlacionais serão utilizados os testes de *Pearson* ou *Spearman*. As variáveis categóricas serão analisadas pelos testes qui-quadrado ou pelo teste exato de Fisher.

As análises estatísticas serão realizadas por meio do programa estatístico SPSS (*Statistical Package for the Social Sciences*), adotando nível de significância de 5% (p<0,05).

Os dados também serão avaliados através do método de análise da variância para medidas repetidas (ANOVA *for repeated measures*) com o objetivo de verificar simultaneamente a influência dos 2 grupos de estudo (avaliação intergrupos) e das 2 avaliações (avaliação intragrupos) para cada uma das variáveis, afim de obter a estimação do efeito interação grupo x tempo. Caso as variáveis numéricas não apresentarem distribuição normal, serão transformadas em *ranks.*

**5.11 Divulgação dos resultados**

Os resultados do estudo serão divulgados às participantes da pesquisa e à Unicamp. Além disso, os resultados da pesquisa serão encaminhados para publicação em jornais de impacto científico e em congressos nacionais e internacionais, com os devidos créditos aos autores e colaboradores.

**6. ASPECTOS ÉTICOS**

O trabalho seguirá as normas de boas práticas em estudos clínicos envolvendo seres humanos, de acordo com a Resolução n. 466/12 do Conselho Nacional de Saúde, e será previamente aprovado pela Comissão de Pesquisa do Centro de Atenção Integral à Saúde da Mulher – CAISM e pelo Comitê de Ética em Pesquisa da Universidade Estadual de Campinas – UNICAMP.

Todas as participantes receberão duas vias do Termo de Consentimento Livre e Esclarecido (Anexo 10) que visa assegurar seus direitos como participante, sendo que uma via ficará com a participante e a outra com a pesquisadora responsável. Neste momento, ficará claro para a participante que a mesma poderá não querer participar ou retirar sua autorização a qualquer momento, bem como serão explicados os objetivos e metodologia do estudo.

As participantes serão esclarecidas sobre quais serão os benefícios em decorrência de sua participação, tais como: avaliação gratuita e tratamento da frouxidão vaginal via RF, TMAP ou as técnicas associadas.

Elas também serão informadas sobre os exames aos quais serão submetidas bem como a ocorrência de desconfortos em relação aos mesmos. Para os exames de palpação digital e POP-Q, apesar de indolor, será utilizado um gel lubrificante antialérgico para diminuir o desconforto causado pela introdução dos dedos do examinador e da régua graduada.

Serão informadas que a aplicação da radiofrequência é um procedimento indolor, via vaginal com duração de 15 a 20 minutos. No vestíbulo e na abertura vaginal, será aplicada lidocaína spray 10% 3 minutos antes do procedimento para evitar qualquer desconforto. Será então introduzido um espéculo vaginal descartável, e posteriormente será realizada a antissepsia com clorexidina aquosa 0,2%, a limpeza com solução salina estéril 0,9% para remover o conteúdo vaginal excedente com gaze.

Além disso, será explicado à participante sobre a garantia de que sua identidade será mantida em sigilo e nenhuma informação será dada a outras pessoas que não façam parte da equipe de pesquisadores, ressaltando que na divulgação dos resultados desse estudo, seu nome não será citado.

**7. ORÇAMENTO**

O presente estudo será desenvolvido nas dependências da Universidade Estadual de Campinas – UNICAMP/SP, uma vez que a universidade já dispõe de infraestrutura e equipamentos necessários para o desenvolvimento da proposta, como a equipe de fisioterapia e o aparelho de eletromiografia.

A descrição de gastos para custeio dos insumos dos procedimentos está descrita no anexo 10. O restante dos custos será inteiramente subsidiado pelos próprios pesquisadores. O aparelho de Radiofrequência está sendo fornecido pela empresa fabricante Loktal Medical Electronics. É importante ressaltar que a empresa não apresenta influência na redação do projeto e desenho do estudo, nem está remunerando nenhum dos pesquisadores envolvidos direta ou indiretamente na realização desta pesquisa.

**8. PLANO DE ATIVIDADES E CRONOGRAMA**

O estudo será realizado no período de vinte e quatro meses. As fases da pesquisa estão organizadas em cronograma abaixo.


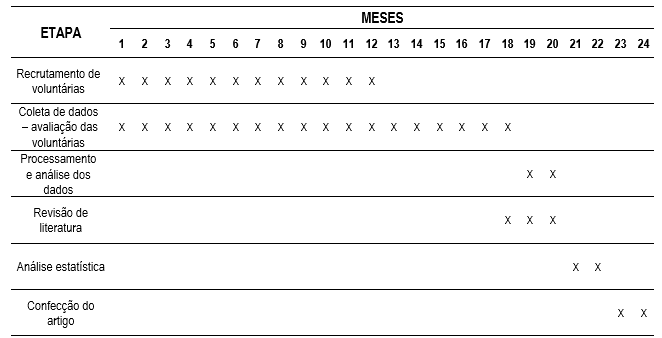
 **Tabela 1** - Cronograma de atividades previstas.

**9. REFERÊNCIAS**

1. Haylen B, De Ridder D, Freeman R, Swift S, Berghmans B, Lee J. International Continence Society. An International Urogynecological Association (IUGA)/International Continence Society (ICS) joint report on the terminology for female pelvic floor dysfunction. *Neurourol Urodyn*. 2010;29(1):4-20.

2. Singh A, Swift S, Khullar V, Digesu GA. Laser vaginal rejuvenation: not ready for prime time. Springer; 2015.

3. Hamori CA. Aesthetic surgery of the female genitalia: labiaplasty and beyond. *Plastic and reconstructive surgery*. 2014;134(4):661-673.

4. Millheiser LS, Pauls RN, Herbst SJ, Chen BH. Radiofrequency treatment of vaginal laxity after vaginal delivery: nonsurgical vaginal tightening. *The journal of sexual medicine*. 2010;7(9):3088-3095.

5. Pauls RN, Fellner AN, Davila GW. Vaginal laxity: a poorly understood quality of life problem; a survey of physician members of the International Urogynecological Association (IUGA). *International urogynecology journal*. 2012;23(10):1435-1448.

6. Moore R, Miklos J, Chinthakanan O. Evaluation of sexual function outcomes in women undergoing vaginal rejuvenation/vaginoplasty procedures for symptoms of vaginal laxity/decreased vaginal sensation utilizing validated sexual function questionnaire (PISQ-12). *Surgical technology international*. 2014;24:253-260.

7. Dietz HP, Stankiewicz M, Atan IK, Ferreira CW, Socha M. Vaginal laxity: what does this symptom mean? *International urogynecology journal*. 2018:1-6.

8. Berman L, Windecker MA. The relationship between women’s genital self-image and female sexual function: A national survey. *Current Sexual Health Reports*. 2008;5(4):199-207.

9. Dietz HP, Wilson PD, Milsom I. Maternal birth trauma: why should it matter to urogynaecologists? *Current Opinion in Obstetrics and Gynecology*. 2016;28(5):441-448.

10. Sekiguchi Y, Utsugisawa Y, Azekosi Y, et al. Laxity of the vaginal introitus after childbirth: nonsurgical outpatient procedure for vaginal tissue restoration and improved sexual satisfaction using low-energy radiofrequency thermal therapy. *Journal of Women's Health*. 2013;22(9):775-781.

11. Zielinski R, Miller J, Low LK, Sampselle C, DeLancey JO. The relationship between pelvic organ prolapse, genital body image, and sexual health. *Neurourology and urodynamics*. 2012;31(7):1145-1148.

12. Barrett G, Pendry E, Peacock J, Victor C, Thakar R, Manyonda I. Women's sexual health after childbirth. *Bjog*. Feb 2000;107(2):186-95.

13. Griffiths A, Watermeyer S, Sidhu K, Amso N, Nix B. Female genital tract morbidity and sexual function following vaginal delivery or lower segment caesarean section. *Journal of obstetrics and gynaecology*. 2006;26(7):645-649.

14. Aslan E, Fynes M. Female sexual dysfunction. *International Urogynecology Journal*. 2008;19(2):293-305.

15. Yang SH, Yang JM, Wang KH, Huang WC. Biologic correlates of sexual function in women with stress urinary incontinence. *The journal of sexual medicine*. 2008;5(12):2871-2879.

16. Graziottin A, Leiblum SR. Biological and psychosocial pathophysiology of female sexual dysfunction during the menopausal transition. *The Journal of Sexual Medicine*. 2005;2:133-145.

17. Faisal-Cury A, Menezes PR, Quayle J, Matijasevich A, Diniz SG. The relationship between mode of delivery and sexual health outcomes after childbirth. *J Sex Med*. May 2015;12(5):1212-20. doi:10.1111/jsm.12883

18. Klein MC, Gauthier RJ, Robbins JM, et al. Relationship of episiotomy to perineal trauma and morbidity, sexual dysfunction, and pelvic floor relaxation. *Am J Obstet Gynecol*. Sep 1994;171(3):591-8.

19. Signorello LB, Harlow BL, Chekos AK, Repke JT. Postpartum sexual functioning and its relationship to perineal trauma: a retrospective cohort study of primiparous women. *Am J Obstet Gynecol*. Apr 2001;184(5):881-8; discussion 888-90. doi:10.1067/mob.2001.113855

20. Abraham S. Recovery after childbirth. *Med J Aust*. Apr 02 1990;152(7):387.

21. Clarkson J, Newton C, Bick D, et al. Achieving sustainable quality in maternity services - using audit of incontinence and dyspareunia to identify shortfalls in meeting standards. *BMC Pregnancy Childbirth*. 2001;1(1):4.

22. Abdool Z, Shek KL, Dietz HP. The effect of levator avulsion on hiatal dimension and function. *American journal of obstetrics and gynecology*. 2009;201(1):89. e1-89. e5.

23. Dietz HP. PELVIC FLOOR ASSESSMENT. *Fetal and Maternal Medicine Review*. 2009;20(1):49-66.

24. Lien K-C, Mooney B, DeLancey JO, Ashton-Miller JA. Levator ani muscle stretch induced by simulated vaginal birth. *Obstetrics and gynecology*. 2004;103(1):31.

25. Svabik K, Shek K, Dietz H. How much does the levator hiatus have to stretch during childbirth? *BJOG: An International Journal of Obstetrics & Gynaecology*. 2009;116(12):1657-1662.

26. Brooks SV, Zerba E, Faulkner JA. Injury to muscle fibres after single stretches of passive and maximally stimulated muscles in mice. *The Journal of physiology*. 1995;488(2):459-469.

27. Dietz HP, Lanzarone F. Levator trauma after vaginal delivery. *Obstetrics and Gynecology*. Oct 2005;106(4):707-712. doi:10.1097/01.aog.0000178779.62181.01

28. Kearney R, Miller JM, Ashton-Miller JA, DeLancey JO. Obstetric factors associated with levator ani muscle injury after vaginal birth. *Obstet Gynecol*. Jan 2006;107(1):144-9. doi:10.1097/01.AOG.0000194063.63206.1c

29. Dietz HP, Steensma AB. The prevalence of major abnormalities of the levator ani in urogynaecological patients. *Bjog*. Feb 2006;113(2):225-30. doi:10.1111/j.1471-0528.2006.00819.x

30. Shek K, Dietz H. The effect of vaginal childbirth on levator hiatal dimensions. *International Urogynecology Journal*. Sep 2008;19:S130-S130.

31. Shek KL, Dietz HP. Intrapartum risk factors for levator trauma. *Bjog-an International Journal of Obstetrics and Gynaecology*. Nov 2010;117(12):1485-1492. doi:10.1111/j.1471-0528.2010.02704.x

32. Shek KL, Pirpiris A, Dietz HP. Does levator avulsion increase urethral mobility? *Eur J Obstet Gynecol Reprod Biol*. Dec 2010;153(2):215-9. doi:10.1016/j.ejogrb.2010.07.036

33. Jung S-A, Pretorius DH, Padda BS, et al. Vaginal high-pressure zone assessed by dynamic 3-dimensional ultrasound images of the pelvic floor. *American journal of obstetrics and gynecology*. 2007;197(1):52. e1-52. e7.

34. Longcope C. Metabolic clearance and blood production rates of estrogens in postmenopausal women. *American Journal of Obstetrics and Gynecology*. 1971;111(6):778-781.

35. Tan O, Bradshaw K, Carr BR. Management of vulvovaginal atrophy-related sexual dysfunction in postmenopausal women: an up-to-date review. *Menopause*. 2012;19(1):109-117.

36. Cody JD, Jacobs ML, Richardson K, Moehrer B, Hextall A. Oestrogen therapy for urinary incontinence in post‐menopausal women. *Cochrane Database of Systematic Reviews*. 2012;(10)

37. Clobes A, DeLancey JO, Morgan DM. Urethral circular smooth muscle in young and old women. *American journal of obstetrics and gynecology*. 2008;198(5):587. e1-587. e5.

38. Campbell P, Krychman M, Gray T, et al. Self-Reported Vaginal Laxity—Prevalence, Impact, and Associated Symptoms in Women Attending a Urogynecology Clinic. *The journal of sexual medicine*. 2018;15(11):1515-1517.

39. Krychman ML. Vaginal laxity issues, answers and implications for female sexual function. *The journal of sexual medicine*. 2016;13(10):1445-1447.

40. Krychman M, Rowan CG, Allan BB, et al. Effect of single-treatment, surface-cooled radiofrequency therapy on vaginal laxity and female sexual function: the VIVEVE I randomized controlled trial. *The journal of sexual medicine*. 2017;14(2):215-225.

41. Chen L, Ashton-Miller JA, Hsu Y, DeLancey J. Interaction among apical support, levator ani impairment, and anterior vaginal wall prolapse. *Obstetrics and gynecology*. 2006;108(2):324-332.

42. Stein TA, DeLancey JO. Structure of the perineal membrane in females: gross and microscopic anatomy. *Obstetrics and gynecology*. 2008;111(3):686.

43. Corsini-Munt S, Bergeron S, Rosen NO, et al. A comparison of cognitive-behavioral couple therapy and lidocaine in the treatment of provoked vestibulodynia: Study protocol for a randomized clinical trial. Article. *Trials*. 2014;15(1)506. doi:10.1186/1745-6215-15-506.

44. Cundiff GW, Fenner D. Evaluation and treatment of women with rectocele: focus on associated defecatory and sexual dysfunction. *Obstetrics & Gynecology*. 2004;104(6):1403-1421.

45. Lewicky-Gaupp C, Fenner DE, Delancey JO. Posterior vaginal wall repair: Does anatomy matter? *Contemporary Ob/Gyn*. 2009;54(10):44-49.

46. Ghoniem G, Stanford E, Kenton K, et al. Evaluation and outcome measures in the treatment of female urinary stress incontinence: International Urogynecological Association (IUGA) guidelines for research and clinical practice. *International Urogynecology Journal*. 2008;19(1):5-33.

47. Haylen BT, Maher CF, Barber MD, et al. Erratum to: An International Urogynecological Association (IUGA)/International Continence Society (ICS) joint report on the terminology for female pelvic organ prolapse (POP). *International urogynecology journal*. 2016;27(4):655-684.

48. Laycock J. Female pelvic floor assessment: the Laycock ring of continence. *J Natl Women Health Group Aust Physiother Assoc*. 1994:40-51.

49. Practice CoG. ACOG Committee Opinion No. 378: Vaginal" rejuvenation" and cosmetic vaginal procedures. *Obstetrics and gynecology*. 2007;110(3):737.

50. Shafik A. The role of the levator ani muscle in evacuation, sexual performance and pelvic floor disorders. *International Urogynecology Journal*. 2000;11(6):361-376.

51. Bø K, Talseth T, Vinsnes A. Randomized controlled trial on the effect of pelvic floor muscle training on quality of life and sexual problems in genuine stress incontinent women. *Acta obstetricia et gynecologica Scandinavica*. 2000;79(7):598-603.

52. Goldfinger C, Pukall CF, Gentilcore-Saulnier E, McLean L, Chamberlain S. PAIN: A Prospective Study of Pelvic Floor Physical Therapy: Pain and Psychosexual Outcomes in Provoked Vestibulodynia. *The journal of sexual medicine*. 2009;6(7):1955-1968.

53. Dumoulin C, Hay‐Smith J, Habée‐Séguin GM, Mercier J. Pelvic floor muscle training versus no treatment, or inactive control treatments, for urinary incontinence in women: a short version Cochrane systematic review with meta‐analysis. *Neurourology and urodynamics*. 2015;34(4):300-308.

54. Tadir Y, Gaspar A, Lev‐Sagie A, et al. Light and energy based therapeutics for genitourinary syndrome of menopause: consensus and controversies. *Lasers in surgery and medicine*. 2017;49(2):137-159.

55. Elser DM, Mitchell GK, Miklos JR, et al. Nonsurgical transurethral collagen denaturation for stress urinary incontinence in women: 18‐month results from a prospective long‐term study. *Neurourology and urodynamics*. 2010;29(8):1424-1428.

56. Dillon B, Dmochowski R. Radiofrequency for the treatment of stress urinary incontinence in women. *Current urology reports*. 2009;10(5):369-374.

57. Hodgkinson DJ. Clinical applications of radiofrequency: nonsurgical skin tightening (thermage). *Clinics in plastic surgery*. 2009;36(2):261-268.

58. Dunbar SW, Goldberg DJ. Radiofrequency in Cosmetic Dermatology: An Update. *Journal of drugs in dermatology: JDD*. 2015;14(11):1229-1238.

59. Coad J, Vos J, Curtis A, Krychman M. safety And Mechanisms Of Action Supporting Nonablative Radiofrequency Thermal Therapy For Vaginal Introitus Laxity Occurring In Women After Childbirth: Histological Study In The Sheep Vaginal Model: poster# 16. *The Journal of Sexual Medicine*. 2013;10:175.

60. Rosen R, Brown C, Heiman J, et al. The Female Sexual Function Index (FSFI): a multidimensional self-report instrument for the assessment of female sexual function. *J Sex Marital Ther*. Apr-Jun 2000;26(2):191-208. doi:10.1080/009262300278597

61. Mira TA, Giraldo PC, Yela DA, Benetti-Pinto CL. Effectiveness of complementary pain treatment for women with deep endometriosis through Transcutaneous Electrical Nerve Stimulation (TENS): randomized controlled trial. *European Journal of Obstetrics & Gynecology and Reproductive Biology*. 2015;194:1-6.

62. Tamanini JTN, Almeida FG, Girotti ME, Riccetto CL, Palma PC, Rios LAS. The Portuguese validation of the International Consultation on Incontinence Questionnaire—Vaginal Symptoms (ICIQ-VS) for Brazilian women with pelvic organ prolapse. *International Urogynecology Journal*. 2008;19(10):1385-1391.

63. DeRogatis L, Clayton A, Lewis-D'Agostino D, Wunderlich G, Fu Y. Validation of the female sexual distress scale-revised for assessing distress in women with hypoactive sexual desire disorder. *The journal of sexual medicine*. 2008;5(2):357-364.

64. Tamanini JT, Dambros M, D'Ancona CA, Palma PC, Rodrigues Netto N, Jr. [Validation of the "International Consultation on Incontinence Questionnaire -- Short Form" (ICIQ-SF) for Portuguese]. *Rev Saude Publica*. Jun 2004;38(3):438-44. Validacao para o portugues do "International Consultation on Incontinence Questionnaire -- Short Form" (ICIQ-SF). doi:/S0034-89102004000300015

65. Dietz HP. Pelvic floor ultrasound: a review. *American Journal of Obstetrics and Gynecology*. Apr 2010;202(4):321-334. doi:10.1016/j.ajog.2009.08.018

66. Balica A, Wald-Spielman D, Schertz K, Egan S, Bachmann G. Assessing the thickness of the vaginal wall and vaginal mucosa in pre-menopausal versus post-menopausal women by transabdominal ultrasound: A feasibility study. *Maturitas*. Aug 2017;102:69-72. doi:10.1016/j.maturitas.2017.02.017

67. Panayi DC, Digesu GA, Tekkis P, Fernando R, Khullar V. Ultrasound measurement of vaginal wall thickness: a novel and reliable technique. *Int Urogynecol J*. Oct 2010;21(10):1265-70. doi:10.1007/s00192-010-1183-4

68. Kamilos MF, Borrelli CL. New therapeutic option in genitourinary syndrome of menopause: pilot study using microablative fractional radiofrequency. *Einstein (São Paulo)*. 2017;15(4):445-451.

69. Dumoulin C, Morin M, Mayrand MH, Tousignant M, Abrahamowicz M. Group physiotherapy compared to individual physiotherapy to treat urinary incontinence in aging women: study protocol for a randomized controlled trial. *Trials*. Nov 16 2017;18(1):544. doi:10.1186/s13063-017-2261-4

70. Bø K, Talseth T, Holme I. Single blind, randomised controlled trial of pelvic floor exercises, electrical stimulation, vaginal cones, and no treatment in management of genuine stress incontinence in women. *Bmj*. Feb 20 1999;318(7182):487-93. doi:10.1136/bmj.318.7182.487

71. Thiel Rdo R, Dambros M, Palma PC, Thiel M, Riccetto CL, Ramos Mde F. [Translation into Portuguese, cross-national adaptation and validation of the Female Sexual Function Index]. *Rev Bras Ginecol Obstet*. Oct 2008;30(10):504-10. Traducao para portugues, adaptacao cultural e validacao do Female Sexual Function Index.

72. Wiegel M, Meston C, Rosen R. The Female Sexual Function Index (FSFI): Cross-validation and development of clinical cutoff scores. *Journal of Sex & Marital Therapy*. Jan-Feb 2005;31(1):1-20. doi:10.1080/00926230590475206

73. Bump RC, Mattiasson A, Bø K, et al. The standardization of terminology of female pelvic organ prolapse and pelvic floor dysfunction. *American journal of obstetrics and gynecology*. 1996;175(1):10-17.

74. Garnham AP, Rojas RG, Shek KL, Dietz HP. Predicting levator avulsion from ICS POP-Q findings. *International Urogynecology Journal*. 2014:1-5.

75. Ferreira CHJ, Barbosa PB, de Oliveira Souza F, Antônio FI, Franco MM, Bø K. Inter-rater reliability study of the modified Oxford Grading Scale and the Peritron manometer. *Physiotherapy*. 2011;97(2):132-138.

76. Cyr MP, Kruger J, Wong V, Dumoulin C, Girard I, Morin M. Pelvic floor morphometry and function in women with and without puborectalis avulsion in the early postpartum period. *Am J Obstet Gynecol*. Mar 2017;216(3):274.e1-274.e8. doi:10.1016/j.ajog.2016.11.1049

**10. DIVULGAÇÃO DA PESQUISA**

A pesquisa será divulgada em mídias sociais, em jornais, em rádios e em programas de televisão com o objetivo de auxiliar no processo de recrutamento de pacientes voluntárias, mediante a leitura do texto, reportagens ou divulgação do texto impresso ou em postagens em mídias sociais.

PESQUISA FROUXIDÃO VAGINAL

A Universidade Estadual de Campinas – UNICAMP convida mulheres maiores de 18 anos, com pelo menos um parto normal (via vaginal) e com queixa de frouxidão vaginal (vagina larga) para participarem da Pesquisa Frouxidão Vaginal. A pesquisa será realizada no Caism - Hospital da Mulher J. A. Pinotti, com o apoio da equipe de Uroginecologia e visa avaliar duas opções de tratamentos gratuitos – a fisioterapia e a radiofrequência.

A fisioterapia tem um papel importante na função sexual feminina com o fortalecimento dos músculos que dão suporte à vagina.

A radiofrequência é um tratamento inovador que atua nas camadas vaginais proporcionando maior resistência à vagina. A radiofrequência utiliza alta tecnologia por meio de tratamento via vaginal e indolor.

Tire suas dúvidas e se informe pelo telefone (WhatsApp) 19 98176 7113 ou por e-mail: pesquisafrouxidaovaginal@gmail.com

Fique atenta:

A frouxidão vaginal ou vagina larga é definida como excesso de flacidez vaginal, ocorrendo em qualquer idade e podendo afetar a vida sexual das mulheres e seus relacionamentos.

**ANEXO 1 – Vaginal Laxity Questionnaire (VLQ)**

Como você avaliaria seu nível atual de frouxidão vaginal? ou frouxidão durante a relação sexual? ESCORE ______

1- Muito frouxa; 2- Moderadamente frouxa; 3- Ligeiramente frouxa; 4- Nem frouxa nem apertada; 5- Ligeiramente apertada; 6- Moderadamente apertada; 7- Muito apertada

**ANEXO 2 - Female Sexual Function Index (FSFI)**

**Female Sexual Function Index (FSFI)**

Estas perguntas são sobre seus sentimentos e respostas sexuais nas últimas QUATRO SEMANAS. Por favor, responda às seguintes perguntas da forma mais clara e honesta possível. Suas respostas serão mantidas em completo sigilo. As definições (explicações) que seguem são aplicadas para responder o questionário: Atividade sexual: pode incluir carícias, estimulação sexual preliminar, masturbação e coito vaginal. Relação sexual é definida como a penetração (entrada) do pênis na vagina. Estimulação sexual: inclui estimulação sexual preliminar com o parceiro, autoerotismo (masturbação) ou fantasia sexual.

PARA CADA ITEM, MARQUE APENAS UMA RESPOSTA

O desejo ou interesse sexual é um sentimento que abrange a vontade de ter uma experiência sexual, a receptividade às iniciativas sexuais do parceiro, e pensamentos ou fantasias sobre o ato sexual.

1. Durante as últimas 4 semanas, com que frequência você sentiu desejo ou interesse sexual?

(5 ) Sempre ou quase sempre

(4 ) Muitas vezes (mais da metade do tempo)

(3 ) Às vezes (aproximadamente a metade do tempo)

(2 ) Poucas vezes (menos do que a metade do tempo)

(1 ) Nunca ou quase nunca

1. Durante as últimas 4 semanas, como você classificaria seu nível (grau) de desejo ou interesse sexual?

(5 ) Muito alto

(4 ) Alto

(3 ) Moderado

(2 ) Baixo

(1 ) Muito baixo ou nenhum

A excitação sexual é uma sensação com aspectos físicos e mentais. Pode aparecer uma sensação de calor ou de vibração na genitália, lubrificação (umidade), ou contrações musculares.

1. Durante as últimas 4 semanas, com que frequência você se sentiu excitada durante o ato ou atividade sexual?

(0 ) Sem atividade sexual

(5 ) Sempre ou quase sempre

(4 ) Muitas vezes (mais da metade do tempo)

(3 ) Algumas vezes ( metade das vezes)

(2 ) Poucas vezes (menos da metade do tempo)

(1 ) Nunca ou quase nunca

1. Durante as últimas 4 semanas, como você classificaria seu nível (grau) de excitação sexual durante a atividade sexual?

(0 )Sem atividade sexual

(5 ) Muito alto

(4 ) Alto

(3 ) Moderado

(2 ) Baixo

(1 ) Muito baixo ou nenhum

1. Durante as últimas 4 semanas, qual foi seu grau de confiança sobre sentir-se excitada durante a atividade sexual?

(0 ) Sem atividade sexual

(5 ) Altíssima confiança

(4 ) Alta confiança

(3 ) Moderada confiança

(2 ) Baixa confiança

(1 ) Baixíssima ou nenhuma confiança

1. Durante as últimas 4 semanas, com que frequência você ficou satisfeita com seu nível (grau) de excitação durante a atividade sexual?

(0 ) Sem atividade sexual

(5 ) Sempre ou quase sempre

(4 ) Muitas vezes (mais da metade do tempo)

(3 ) Algumas vezes (aproximadamente a metade do tempo)

(2 ) Poucas vezes (menos da metade do tempo)

(1 ) Nunca ou quase nunca

1. Durante as últimas 4 semanas, com que frequência você ficou lubrificada ("molhada") durante a atividade sexual?

(0 ) Sem atividade sexual

(5 ) Sempre ou quase sempre

(4 ) Muitas vezes (mais da metade do tempo)

(3 ) Algumas vezes (aproximadamente a metade do tempo)

(2 ) Poucas vezes (menos da metade do tempo)

(1 ) Nunca ou quase nunca

1. Durante as últimas 4 semanas, qual foi o grau de dificuldade para ficar lubrificada ("molhada") durante a atividade sexual?

(0 ) Sem atividade sexual

(1 ) Extremamente difícil ou impossível

(2 ) Muito difícil

(3 ) Difícil

(4 ) Pouco difícil

(5 ) Nada difícil

1. Durante as últimas 4 semanas, com que frequência você manteve sua lubrificação até o final da atividade sexual?

(0 ) Sem atividade sexual

(5 ) Sempre ou quase sempre

(4 ) Muitas vezes (mais da metade do tempo)

(3 ) Algumas vezes (aproximadamente a metade do tempo)

(2 ) Poucas vezes (menos da metade do tempo)

(1 ) Nunca ou quase nunca

1. Durante as últimas 4 semanas, qual foi o grau de dificuldade para manter sua lubrificação até terminar a atividade sexual?

(0 ) Sem atividade sexual

(1 ) Extremamente difícil ou impossível

(2 ) Muito difícil

(3 ) Difícil

(4 ) Pouco Difícil

(5 ) Nada Difícil

1. Durante as últimas 4 semanas, na atividade sexual ou quando sexualmente estimulada, com que frequência você atingiu o orgasmo (clímax)?

(0 ) Sem atividade sexual

(5 ) Sempre ou quase sempre

(4 ) Muitas vezes (mais da metade do tempo)

(3 ) Algumas vezes (aproximadamente a metade do tempo)

(2 ) Poucas vezes (menos da metade do tempo)

(1 ) Nunca ou quase nunca

1. Durante as últimas 4 semanas, na atividade sexual ou quando sexualmente estimulada, qual foi o grau de dificuldade para atingir o orgasmo (clímax)?

(0 ) Sem atividade sexual

(1 ) Extremamente difícil ou impossível

(2 ) Muito difícil

(3 ) Difícil

(4 ) Pouco Difícil

(5 ) Nada Difícil

1. Durante as últimas 4 semanas, qual foi o grau de satisfação com sua habilidade de chegar ao orgasmo (clímax) durante a atividade sexual?

(0 ) Sem atividade sexual

(5 ) Muito satisfeita

(4 ) Moderadamente satisfeita

(3 ) Indiferente

(2 ) Moderadamente insatisfeita

(1 ) Muito insatisfeita

1. Durante as últimas 4 semanas, qual foi o grau de satisfação com a quantidade de envolvimento emocional entre você e seu parceiro durante a atividade sexual?

(0 ) Sem atividade sexual

(5 ) Muito satisfeita

(4 ) Moderadamente satisfeita

(3 ) Indiferente

(2 ) Moderadamente insatisfeita

(1 ) Muito insatisfeita

1. Durante as últimas 4 semanas, qual foi o grau de satisfação na relação sexual com seu parceiro?

(5 ) Muito satisfeita

(4 ) Moderadamente satisfeita

(3 ) Indiferente

(2 ) Moderadamente insatisfeita

(1 ) Muito insatisfeita

1. Durante as últimas 4 semanas, de forma geral, qual foi o grau de satisfação com sua vida sexual?

(5 ) Muito satisfeita

(4 ) Moderadamente satisfeita

(3 ) Indiferente

(2 ) Moderadamente insatisfeita

(1 ) Muito insatisfeita

1. Durante as últimas 4 semanas, com que frequência você sentiu desconforto ou dor durante a penetração vaginal?

(0 ) Não houve tentativa de penetração

(1 ) Sempre ou quase sempre

(2 ) Muitas vezes (mais da metade do tempo)

(3 ) Algumas vezes (aproximadamente a metade do tempo)

(4 ) Poucas vezes (menos da metade do tempo)

(5 ) Nunca ou quase nunca

1. Durante as últimas 4 semanas, com que frequência você sentiu desconforto ou dor após a penetração vaginal?

(0 ) Não houve tentativa de penetração

(1 ) Sempre ou quase sempre

(2 ) Muitas vezes (mais da metade do tempo)

(3 ) Algumas vezes (aproximadamente a metade do tempo)

(4 ) Poucas vezes (menos da metade do tempo)

(5 ) Nunca ou quase nunca

1. Durante as últimas 4 semanas, como você classificaria seu grau (nível) de desconforto ou dor durante ou após a penetração vaginal?

(0 ) Não houve tentativa de penetração

(1 ) Altíssimo

(2 ) Alto

(3 ) Moderado

(4 ) Baixo

(5 ) Baixíssimo ou nenhum

**ESCORE E ANÁLISE PELO PESQUISADOR:**

| Domínio | Questões | Pontuação | Fator | Pontuação Mínima | Pontuação Máxima | Resultado |
| --- | --- | --- | --- | --- | --- | --- |
| Desejo | 1,2 | 1 - 5 | 0.6 | 1.2 | 6.0 |  |
| Excitação | 3,4,5,6 | 0 - 5 | 0.3 | 0 | 6.0 |  |
| Lubrificação | 7,8,9,10 | 0 - 5 | 0.3 | 0 | 6.0 |  |
| Orgasmo | 11,12,13 | 0 - 5 | 0.4 | 0 | 6.0 |  |
| Satisfação | 14,15,16 | 0 (ou 1)- 5 | 0.4 | 0.8 | 6.0 |  |
| Dor | 17,18,19 | 0 -5 | 0.4 | 0 | 6.0 |  |
| Total |  |  |  | 2.0 | 36.0 |  |

Nota de Corte: 26,55

**ANEXO 3- Escala de Dispareunia de Profundidade (EDP), baseada na Escala de Marinoff**

Sobre dor na relação sexual

| Não possuo relação sexual |  | Não |
| --- | --- | --- |
| Ausência de dor na relação sexual |  | 0 |
| Dor leve, que não obriga a interromper a relação sexual |  | 1 |
| Dor moderada, que dificulta, mas não obriga a interromper a relação sexual |  | 2 |
| Dor intensa, que obriga a interromper a relação sexual |  | 3 |

**ANEXO 4 - International Consultation on Incontinence Questionnaire - Vaginal Symptoms (ICIQ-VS)**

1.    Você percebe uma dor em pressão ou peso no seu abdômen inferior (pé da barriga)?

| Nunca |  | 0 |
| --- | --- | --- |
| Ocasionalmente |  | 1 |
| Às vezes |  | 2 |
| Na maior parte do tempo |  | 3 |
| O tempo todo |  | 4 |

O quanto isso incomoda você? Circule um número de 0 (não incomoda) a 10 (incomoda muito). 0       1       2        3 4       5        6 7       8        9 10

2.   Você percebe que sua vagina está dolorida?

| Nunca |  | 0 |
| --- | --- | --- |
| Ocasionalmente |  | 1 |
| Às vezes |  | 2 |
| Na maior parte do tempo |  | 3 |
| O tempo todo |  | 4 |

O quanto isso incomoda você? Circule um número de 0 (não incomoda) a 10 (incomoda muito).

0      1 2      3 4        5 6       7        8 9       10

3.    Você sente que tem uma redução de sensibilidade ou amortecimento na sua vagina ou em volta dela?

| De jeito nenhum |  | 0 |
| --- | --- | --- |
| Um pouco |  | 1 |
| Moderadamente |  | 2 |
| Muito |  | 3 |

O quanto isso incomoda você? Circule um número de 0 (não incomoda) a 10 (incomoda muito).

0       1       2        3 4       5        6 7       8        9 10

4.    Você sente sua vagina muito frouxa ou larga?

| De jeito nenhum |  | 0 |
| --- | --- | --- |
| Um pouco |  | 1 |
| Moderadamente |  | 2 |
| Muito |  | 3 |

O quanto isso incomoda você? Circule um número de 0 (não incomoda) a 10 (incomoda muito).

0       1       2        3 4       5        6 7       8        9 10

5.    Você percebe um “caroço” ou uma “bola” descendo na sua vagina?

| Nunca |  | 0 |
| --- | --- | --- |
| Ocasionalmente |  | 1 |
| Às vezes |  | 2 |
| Na maior parte do tempo |  | 3 |
| O tempo todo |  | 4 |

O quanto isso incomoda você? Circule um número de 0 (não incomoda) a 10 (incomoda muito).

0       1       2        3 4       5        6 7       8        9 10

6.    Você percebe um “caroço” ou uma “bola” saindo de sua vagina de forma que você possa senti-la o vê-la fora dela?

| Nunca |  | 0 |
| --- | --- | --- |
| Ocasionalmente |  | 1 |
| Às vezes |  | 2 |
| Na maior parte do tempo |  | 3 |
| O tempo todo |  | 4 |

O quanto isso incomoda você? Circule um número de 0 (não incomoda) a 10 (incomoda muito).

0       1       2        3 4       5        6 7       8        9 10

7. Você sente que sua vagina é muito seca?

| Nunca |  | 0 |
| --- | --- | --- |
| Ocasionalmente |  | 1 |
| Às vezes |  | 2 |
| Na maior parte do tempo |  | 3 |
| O tempo todo |  | 4 |

O quanto isso incomoda você? Circule um número de 0 (não incomoda) a 10 (incomoda muito).

0       1       2        3 4       5        6 7       8        9 10

8. Você tem que colocar o dedo na sua vagina para ajudar a evacuar (fazer cocô)?

| Nunca |  | 0 |
| --- | --- | --- |
| Ocasionalmente |  | 1 |
| Às vezes |  | 2 |
| Na maior parte do tempo |  | 3 |
| O tempo todo |  | 4 |

O quanto isso incomoda você? Circule um número de 0 (não incomoda) a 10 (incomoda muito).

0       1       2        3 4       5        6 7       8        9 10

9. Você sente que sua vagina é muito apertada?

| Nunca |  | 0 |
| --- | --- | --- |
| Ocasionalmente |  | 1 |
| Às vezes |  | 2 |
| Na maior parte do tempo |  | 3 |
| O tempo todo |  | 4 |

O quanto isso incomoda você? Circule um número de 0 (não incomoda) a 10 (incomoda muito).

0       1       2        3 4       5        6 7       8        9 10

10. Atualmente você tem vida sexual?

0- Sim

1- Não, por causa dos meus sintomas vaginais

2- Não, por outros motivos

11. O seu problema de vagina interfere na sua vida sexual?

| De jeito nenhum |  | 0 |
| --- | --- | --- |
| Um pouco |  | 1 |
| Moderadamente |  | 2 |
| Muito |  | 3 |

O quanto isso incomoda você? Circule um número de 0 (não incomoda) a 10 (incomoda muito).

0       1       2        3 4       5        6 7       8        9 10

12**.** Você sente que o seu relacionamento é afetado pelos sintomas vaginais?

| De jeito nenhum |  | 0 |
| --- | --- | --- |
| Um pouco |  | 1 |
| Moderadamente |  | 2 |
| Muito |  | 3 |

O quanto isso incomoda você? Circule um número de 0 (não incomoda) a 10 (incomoda muito). 0       1       2        3 4       5        6 7       8        9 10

13**.** O quanto você acha que sua vida sexual tem sido prejudicada pelos seus sintomas vaginais?

Circule um número de 0 (não incomoda) a 10 (incomoda muito).

0       1       2        3 4       5        6 7       8        9 10

**ANEXO 5 – The Female Sexual Distress Scale-Revised *–* (FSDSR)**

Marque 0 = nunca; 1 = raramente; 2 = ocasionalmente; 3 = frequentemente; 4 = sempre.

**Quão frequentemente você se sentiu:**

1. Angustiada com sua vida sexual ( 0 ) ( 1 ) ( 2 ) ( 3 ) ( 4 )

2. Infeliz com o seu relacionamento sexual ( 0 ) ( 1 ) ( 2 ) ( 3 ) ( 4 )

3. Culpada por dificuldades sexuais ( 0 ) ( 1 ) ( 2 ) ( 3 ) ( 4 )

4. Frustrada por seus problemas sexuais ( 0 ) ( 1 ) ( 2 ) ( 3 ) ( 4 )

5. Estressada sobre sexo ( 0 ) ( 1 ) ( 2 ) ( 3 ) ( 4 )

6. Inferior por causa de problemas sexuais ( 0 ) ( 1 ) ( 2 ) ( 3 ) ( 4 )

7. Preocupada com sexo ( 0 ) ( 1 ) ( 2 ) ( 3 ) ( 4 )

8. Sexualmente inadequada ( 0 ) ( 1 ) ( 2 ) ( 3 ) ( 4 )

9. Lamenta sua sexualidade ( 0 ) ( 1 ) ( 2 ) ( 3 ) ( 4 )

10. Envergonhada com problemas sexuais ( 0 ) ( 1 ) ( 2 ) ( 3 ) ( 4 )

11. Insatisfeita com a sua vida sexual ( 0 ) ( 1 ) ( 2 ) ( 3 ) ( 4 )

12. Irritada com a sua vida sexual ( 0 ) ( 1 ) ( 2 ) ( 3 ) ( 4 )

13. Incomodada com baixo desejo sexual ( 0 ) ( 1 ) ( 2 ) ( 3 ) ( 4 )

ESCORE:

**ANEXO 6 - International Consultation on Incontinence Questionnaire Urinary Incontinence – Short Form (ICIQ UI-SF)**

- Frequência da perda urinária:

| *Nunca* |  | *0* |
| --- | --- | --- |
| Uma vez por semana ou menos |  | *1* |
| Duas ou três vezes por semana |  | *2* |
| Uma vez ao dia |  | *3* |
| Diversas vezes ao dia |  | *4* |
| O tempo todo |  | *5* |

- Quantidade de urina perdida:

| Nenhuma |  | 0 |
| --- | --- | --- |
| Uma pequena quantidade |  | 2 |
| Uma moderada quantidade |  | 4 |
| Uma grande quantidade |  | 6 |

Em geral, quanto que perder urina interfere em sua vida diária? Circule um número de 0 (não incomoda) a 10 (incomoda muito).

0 1 2 3 4 5 6 7 8 9 10

**ICIQ-SF Score (1+2+3) = _______________**

- Quando você perde urina?

| Nunca |  | 0 |
| --- | --- | --- |
| Perco antes de chegar ao banheiro |  | 1 |
| Perco quando tusso ou espirro |  | 2 |
| Perco quando estou dormindo |  | 3 |
| Perco quando estou fazendo atividades físicas |  | 4 |
| Perco quando terminei de urinar e estou me vestindo |  | 5 |
| Perco sem razão óbvia |  | 6 |
| Perco o tempo todo |  | 7 |

Observação: Escore variável de 0 a 21 pontos. Quanto maior o escore, maior o comprometimento.

**ANEXO 7 – Pelvic Organ Prolapse Quantification** ***–* (POP-Q)**

Estadio 0: Não há prolapso demonstrado.

Estadio I: A maior parte distal do prolapso está a mais de 1 cm acima do nível do hímen.

Estadio II: A porção mais distal do prolapso situa-se entre 1 cm acima do hímen e 1 cm abaixo do hímen.

Estadio III: A porção mais distal do prolapso está mais de 1 cm para além do plano do hímen, mas evertido pelo menos 2 cm a menos que o comprimento vaginal total.

Estadio IV: Eversão completa ou eversão de até 2 cm do comprimento total do trato

| **AVALIAÇÃO DO PROLAPSO: ESTADIO: ________________** | | | | | | |  |  |  |
| --- | --- | --- | --- | --- | --- | --- | --- | --- | --- |
| Aa (+3 -3) _________ |  |  | Ba (+3 -3) _________ |  |  | C __________ cm | |  |  |
| Hg ____________cm | |  | Cp _____________cm | |  | CVT _________cm | |  |  |
| Ap (+3 -3)_________ |  |  | Bp (+3 -3)_________ |  |  | D ____________cm | |  |  |
| **HIPERMOBILIDADE URETRAL:**  □ SIM □ NÃO | | | | | | | | | |

Aa e Ba =parede vaginal anterior

Ap e Bp =parede vaginal posterior

C e D =vagina superiormente

Hg =hiato genital (do centro do meato uretral externo até a margem posterior do hímen

CVT =comprimento vaginal total (comprimento da vagina do fórnice posterior até o hímen quando o ponto C ou D é reduzido para sua posição normal

Cp =corpo perineal (da margem posterior do hímen até orifício anal)

**ANEXO 8 – Escala de Oxford Modificada e Ultrassonografia**

Escala de Oxford modificada por Laycock ^48^:

0: sem função perineal objetiva;

1: esboço de contração muscular;

2: contração de intensidade fraca;

3: contração de intensidade regular e elevação cranial da parede vaginal;

4: contração de intensidade boa e elevação cranial da parede vaginal;

5: contração de intensidade ótima e elevação cranial da parede vaginal;

ESCORE: _______________

Protocolo Morfometria - Ultrassonografia^65, 76^

Medida em repouso:______________________________________________________

**_____________________________________________________________________________**

Medida em Valsalva: ___________________________________________________________

_____________________________________________________________________________

Medida em Contração: __________________________________________________________

_____________________________________________________________________________

Espessura Vaginal:

Abdominal: ___________________________________________________________________

Transvaginal: _________________________________________________________________

**ANEXO 9 - Termo De Consentimento Livre E Esclarecido (TCLE)**
**TERMO DE CONSENTIMENTO LIVRE E ESCLARECIDO**

**Efeito da radiofrequência microablativa fracionada e do treinamento dos músculos do assoalho pélvico no tratamento de mulheres com queixa de frouxidão vaginal: ensaio clínico randomizado**

Gláucia Miranda Varella Pereira; Cássia Raquel Teatin Juliato**;** Lucia Alves da Silva Lara; Luiz Gustavo Oliveira Brito;

**Número do CAAE:**

A senhora está sendo convidada a participar de uma pesquisa. Este documento, chamado Termo de Consentimento Livre e Esclarecido, visa assegurar seus direitos como participante da pesquisa e é elaborado em duas vias, assinadas e rubricadas pelo pesquisador e pelo participante/responsável legal, sendo que uma via deverá ficar com a senhora e outra com o pesquisador.

Por favor, leia com atenção e calma, aproveitando para esclarecer suas dúvidas. Se houver perguntas antes ou mesmo depois de assiná-lo, você poderá esclarecê-las com o pesquisador. Se preferir, pode levar este Termo para casa e consultar seus familiares ou outras pessoas antes de decidir participar. Não haverá nenhum tipo de penalização ou prejuízo se você não aceitar participar ou retirar sua autorização em qualquer momento. Caso retire sua autorização ou não queira participar a senhora será encaminhada para o tratamento convencional presente no serviço.

**Justificativa e objetivos:**

A senhora está sendo convidada a participar de um estudo que procura avaliar dois tipos de tratamento para sua queixa de frouxidão vaginal. Uma opção de tratamento será a fisioterapia, através do treinamento muscular do assoalho pélvico, cujo benefício é fortalecer os músculos da região da vagina. O outro tipo de tratamento é chamado de radiofrequência – é um aparelho que será colocado na região da vagina, cujo objetivo é melhorar a elasticidade vaginal. Para isso, a senhora será sorteada para um grupo de tratamento e permanecerá nesse grupo até o final da proposta de tratamento. Não sabemos qual desses tratamentos é melhor para tratar a sua queixa, por isso estamos realizando este trabalho.

**Procedimentos:**

Participando do estudo a senhora será convidada a:

- responder algumas perguntas gerais como sua idade, peso, número de partos, etc.

_________________________ _____________________________ Página 1 de 4

Rubrica do (a) Pesquisador(a) Rubrica da Participante

-responder questionários sobre perda de urina, prolapsos vaginais, sintomas na vagina e atividade e satisfação sexual, que demorarão em média 15 minutos no total.

-ser submetida a exames físicos para avaliação dos músculos que dão suporte à vagina (via vaginal – toque vaginal e probe vaginal), exame para avaliar o prolapso vaginal (via vaginal com régua graduada) e ultrassom sobre o períneo (sem introduzir na vagina). Os exames físicos demorarão em média 30 - 40 minutos.

Os questionários e todos os parâmetros serão realizados antes do tratamento e 30 dias e 6 meses após o término do tratamento. Um sorteio definirá qual tratamento a senhora fará: radiofrequência isolada ou fisioterapia isolada. A senhora não poderá escolher qual tratamento vai realizar. Se for realizado procedimento de radiofrequência, serão 3 aplicações mensais, indolores e intravaginais. Se for a fisioterapia, serão realizadas sessões individuais 1 vez por semana por 12 semanas. A senhora realizará também o tratamento em casa e receberá as orientações para executá-lo.

**Desconfortos e riscos:**

A senhora poderá se sentir desconfortável em responder as perguntas e em ser examinada ginecologicamente. Não é esperado nenhum incomodo durante ou após a realização da fisioterapia. A radiofrequência é um procedimento indolor, mas pode ocasionar desconforto leve em algumas pacientes durante sua aplicação. A radiofrequência não possui efeito colateral como secreção, sangramento, dor crônica, infecção ou câncer. A senhora não deverá ter relações sexuais 3 dias antes da radiofrequência, assim como não usar pomadas ou cremes intravaginais. Após cada sessão da radiofrequência a senhora não poderá ter relações sexuais por 10 dias.

**Benefícios:**

A senhora terá como benefício o acesso a um tratamento especializado para a frouxidão vaginal, com radiofrequência ou fisioterapia, e em contrapartida contribuirá para um melhor entendimento a respeito dos tratamentos para a frouxidão vaginal. Caso os resultados da pesquisa mostrem que um grupo é melhor que o outro para o tratamento, caso a senhora esteja no grupo que não mostrou esse benefício, a senhora terá o direito de tratar no grupo contrário após o término da pesquisa, se a senhora assim desejar.

**Acompanhamento e assistência:**

Caso haja qualquer problema que não possibilite sua participação no estudo, a senhora será encaminhada para o ambulatório de ginecologia cirúrgica e ou para o setor de fisioterapia do CAISM, mesmo que a senhora não deseje mais participar da pesquisa. A pesquisa não mudará em nada o seu tratamento caso a senhora resolva não participar da pesquisa. A senhora terá o seu acompanhamento garantido, mesmo após o fim da pesquisa, para ser avaliada em caso de queixas que possam estar relacionadas a pesquisa, independente do término da mesma. Em caso de falta às consultas previamente agendadas para a realização dos exames ou procedimentos de radiofrequência ou fisioterapia sem justificativa, a senhora será desligada da pesquisa e outra voluntária será convidada.

__________________________ _____________________________ Página 2 de 4

Rubrica do (a) Pesquisador(a) Rubrica da Participante

**Sigilo e privacidade:**

A senhora tem a garantia de que sua identidade será mantida em sigilo e nenhuma informação será dada a outras pessoas que não façam parte da equipe de pesquisadores. Na divulgação dos resultados desse estudo, o seu nome não será citado. Os resultados desta pesquisa não estarão em seu prontuário médico.

**Ressarcimento e Indenização:**

A senhora não receberá nenhuma ajuda de custo para participar da pesquisa. A pesquisa será realizada durante as sessões de fisioterapia que a senhora teria agendada (rotina de tratamento definida pelo serviço). Desta forma, a senhora não terá gastos extras para participar da pesquisa. A senhora terá direito à indenização em casos de danos diretos e indiretos decorrentes da pesquisa. Não haverá custo para a realização dos exames e tão pouco com o tratamento de radiofrequência (caso seja este o tratamento sorteado). Todos os exames serão realizados no mesmo dia da avaliação ou tratamento fisioterapêutico com objetivo de facilitar seu deslocamento.

**Contato:**

Em caso de dúvidas sobre a pesquisa, a senhora poderá entrar em contato com os pesquisadores Gláucia Varella ou Luiz Gustavo Brito: Rua Alexandre Fleming, 79 Campinas – SP; telefone (WhatsApp) (19) 9 8176 7113.

Em caso de denúncias ou reclamações sobre sua participação e sobre questões éticas do estudo, a senhora poderá entrar em contato com a secretaria do Comitê de Ética em Pesquisa (CEP) da UNICAMP das 08:00hs às 11:30hs e das 13:00hs as 17:30hs na Rua: Tessália Vieira de Camargo, 126; CEP 13083-887 Campinas – SP; telefone (19) 3521-8936 ou (19) 3521-7187; e-mail: [cep@fcm.unicamp.br](mailto:cep@fcm.unicamp.br).

**O Comitê de Ética em Pesquisa (CEP).**

O papel do CEP é avaliar e acompanhar os aspectos éticos de todas as pesquisas envolvendo seres humanos. A Comissão Nacional de Ética em Pesquisa (CONEP), tem por objetivo desenvolver a regulamentação sobre proteção dos seres humanos envolvidos nas pesquisas. Desempenha um papel coordenador da rede de Comitês de Ética em Pesquisa (CEPs) das instituições, além de assumir a função de órgão consultor na área de ética em pesquisas

__________________________ _____________________________ Página 3 de 4

Rubrica do (a) Pesquisador(a) Rubrica da Participante

**Consentimento livre e esclarecido:**

Após ter recebido os esclarecimentos sobre a natureza da pesquisa, seus objetivos, métodos, benefícios previstos, potenciais riscos e o incômodo que esta possa acarretar, aceito participar:

Nome do (a) participante da pesquisa: ________________________________________

_________________________________________________Data: ____/_____/______

(Assinatura do participante da pesquisa ou nome e assinatura do seu RESPONSÁVEL LEGAL)

**Responsabilidade do Pesquisador:**

Asseguro ter cumprido as exigências da resolução 466/2012 CNS/MS e complementares na elaboração do protocolo e na obtenção deste Termo de Consentimento Livre e Esclarecido. Asseguro, também, ter explicado e fornecido uma via deste documento ao participante da pesquisa. Informo que o estudo foi aprovado pelo CEP perante o qual o projeto foi apresentado e pela CONEP, quando pertinente. Comprometo-me a utilizar o material e os dados obtidos nesta pesquisa exclusivamente para as finalidades previstas neste documento ou conforme o consentimento dado pelo participante da pesquisa.

_________________________________________________Data: ____/_____/______.

(Assinatura do pesquisador)

Página 4 de 4

**ANEXO 10 -Orçamento**

| **UNIVERSIDADE ESTADUAL DE CAMPINAS**  **HOSPITAL DA MULHER PROF. DR. JOSÉ ARISTODEMO PINOTTI – CAISM** | | | | | |
| --- | --- | --- | --- | --- | --- |
| **ORÇAMENTO - ESTIMATIVA** | | | | | |
| **TÍTULO DO PROJETO**: Efeito da radiofrequência microablativa fracionada e do treinamento dos músculos do assoalho pélvico no tratamento de mulheres com queixa de frouxidão vaginal: ensaio clínico randomizado  **PESQUISADOR PRINCIPAL**: Prof. Dr. Luiz Gustavo Oliveira Brito  **PESQUISADORA COLABORADORA**: Gláucia Miranda Varella Pereira | | | | | |
| **Material de Consumo** | | | | | |
| Item | Descrição | Unid. | Qde | Valor Unitário | Valor Total |
| 1 | Espéculo | unid | 315 | R$ 1,87 | R$589,05 |
| 2 | Lidocaína Spray 50mL | unid | 10 | R$68,00 | R$680,00 |
| 3 | Clorexidina 100mL | unid | 20 | R$ 4,00 | R$ 80,00 |
| 4 | Solução Salina Estéril | unid | 55 | R$ 5,00 | R$ 275,00 |
| 5 | Gaze | unid | 100 | R$ 1,10 | R$ 110,00 |
| 6 | Gel | Kg | 5 | R$11,00 | R$ 55,00 |
| 7 | Abaixador de Língua | Pacote | 3 | R$ 5,98 | R$ 17,94 |
| **Item Permanente**: Não se Aplica | | | | | |
| **Serviço de Terceiros**: Não se Aplica | | | | | |
| **Diárias**: Não se Aplica | | | | | |
| **Despesas com Transporte:** Não se Aplica | | | | | |
| **Despesas Totais:** R$ 1.806,99 | | | | | |
